# Supplementary material for: Photocobilins integrate B12 and bilin photochemistry for enzyme control
Source: Nat Commun. 2024 Mar 28;15:2740. doi: 10.1038/s41467-024-46995-1 (PMC10979010; doi:10.1038/s41467-024-46995-1)
Supplement: Supplementary file 1 — Supplementary Information [file 41467_2024_46995_MOESM1_ESM.docx]

**Supplementary information**

**Photocobilins integrate B12 and bilin photochemistry for enzyme control**

Shaowei Zhang^1, 2,^ *, Laura N. Jeffreys^1^, Harshwardhan Poddar^1^, Yuqi Yu^1^, Chuanyang Liu^2^, Kaylee Patel^1^, Linus O. Johannissen^1^, Lingyun Zhu^2^, Matthew J. Cliff^1^, Cunyu Yan^1^, Giorgio Schirò^3^, Martin Weik^3^, Michiyo Sakuma^1^, Colin W. Levy^1^, David Leys^1^, Derren J. Heyes^1,^ *, Nigel S. Scrutton^1,^ *

**Supplementary Figures:**


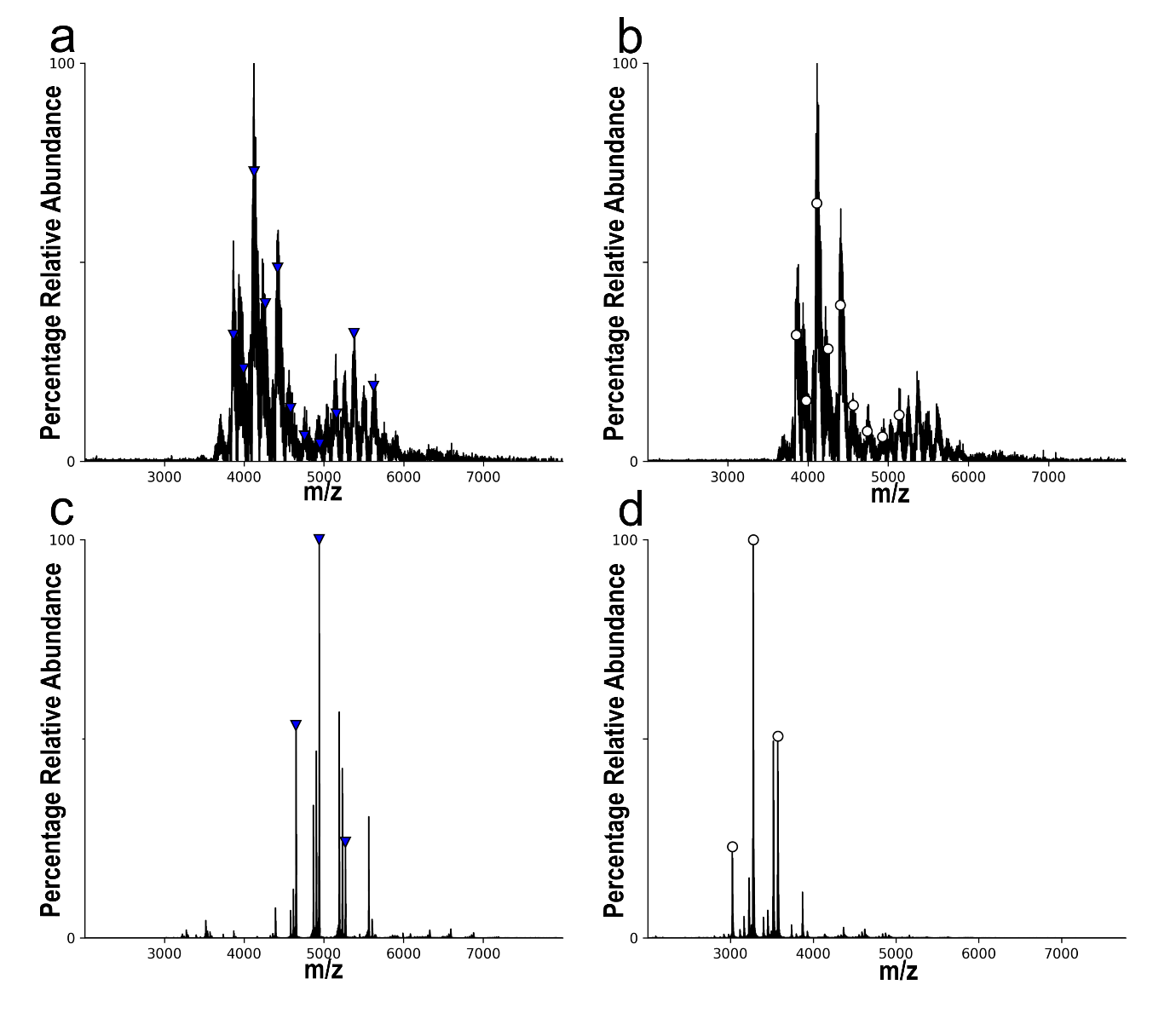


**Supplementary Figure 1: Native mass spectrometry was undertaken to determine the mass of *Sas*Pcob and *Ab*DPcob under dark (blue triangles) and light conditions (white circles).** For all spectra multiple states were observed, corresponding to the protein with and without biliverdin bound. The labelled peaks correspond to the correct mass of the protein with adenosylcobalamin and biliverdin bound. Full length *Ab*DPcob protein had a predominant species with a mass at 123441 (panel a) in the dark and 123191 in the light (panel b) corresponding to the loss of two adenosyl moieties. These unusual spectra appear to show the presence of multiple oligomerisation states that could not be fully elucidated by native MS. *Sas*Pcob had a predominant species with a mass at 79059 in the dark (panel c) and 39278 in the light (panel d) corresponding to a conformational change from a dimer to a monomer.

**Supplementary Figure 2. Size exclusion chromatography-multi-angle light scattering (SEC-MALS) analysis of *Ab*DGC and *Ab*Pcob.** SEC-MALS chromatograms are shown for the following samples: *Ab*DGC and *Ab*Pcob with different concentration, **a** (1 mg/mL), **b** (5 mg/mL), **c** and **d** (1 mg/mL). *Ab*Pcob protein was run through SEC-MALS under dark (**c**) and light (**d**) condition. **e-h**, *Ab*DGC protein was mixed with *Ab*BG at different concentration and run through the SEC-MALS under dark (**e, g**) and light (**f, h**) condition. **e** and **f**, *Ab*DGC mixed with *Ab*Pcob at equal molar ratio. **g** and **h**, *Ab*DGC mixed with 5 times of *Ab*Pcob. Source data are provided in Source Data file.

**Supplementary Figure 3. Absorbance spectra of photocobilins titration assay.** a, AdoCbl with increasing concentration of *Sas*Pcob. b, BV with increasing concentration of *Sas*Pcob. c, BV with increasing concentration of *Sas*Pcob with AdoCbl bound. Source data are provided in Source Data file.

**Supplementary Figure 4. Absorbance spectra of photocobilins with BV and AdoCbl bound**. **a-b**, difference spectra of *Ab*DPcob with both AdoCbl and BV bound after illumination with green (**a**) or red (**b**) light. **c-d,** difference spectra of *Sas*Pcob with BV bound before and after light illumination with red (**c**) or green (**d**) light. Source data are provided in Source Data file.


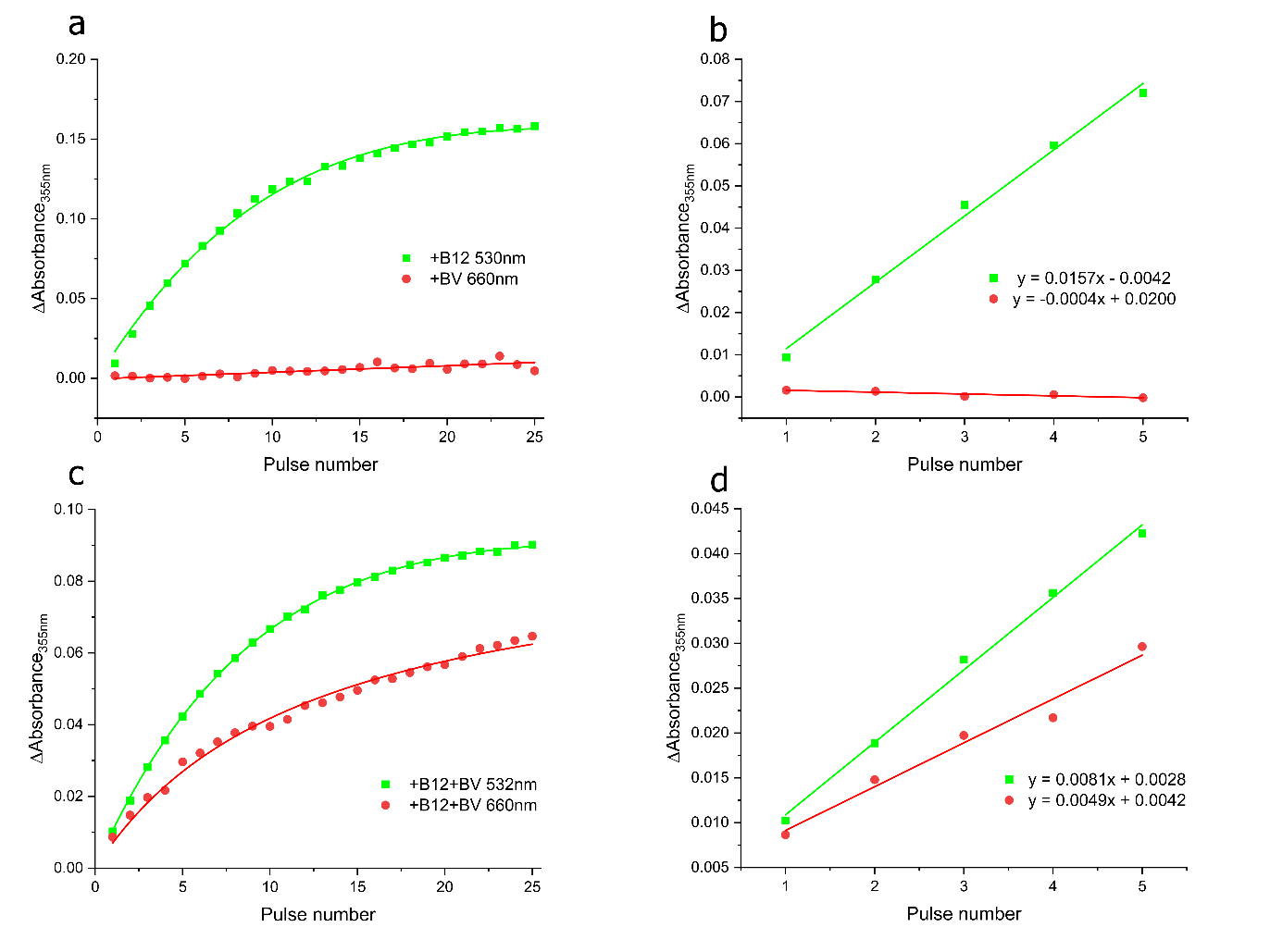


**Supplementary Figure 5. Relative efficiency of conversion to light state upon illumination with green and red light**. The absorbance change at 355nm of *Sas*Pcob samples containing one (**a**, **b**) or both (**c**, **d**) chromophores was measured to calculate the level of conversion to the light state. Samples were illuminated with 100 ms pulses of either 532 nm or 660 nm LED light (150 µmoles m^-2^ s^-1^). Data were fitted to a straight line with the slope used as an estimate of the relative quantum yield for the photoconversion. Source data are provided in Source Data file.

**Supplementary Figure 6. Electron density maps of Pcobs crystals and crystal packing of *Sas*Pcob in dark and light state. a-c,** Electron density maps of main residues around B_12_ and BV in *Ab*Pcob and *Sas*Pcob crystals. **a**, *Ab*Pcob in dark state **b**, *Sas*Pcob in dark state **c**, *Sas*Pcob in light state. Main residues around the chromophore are shown as green sticks and B_12_, BV as magenta, yellow lines respectively. 2Fo−Fc map contoured at 1σ, color as blue and Fo−Fc map contoured at 3σ, color as red. **d-f,** Electron density maps (2Fo−Fc map contoured at 1σ, color as blue and Fo−Fc map contoured at 3σ, color as red) of AdoCbl and BV chromophore in *Ab*Pcob and *Sas*Pcob crystals. **d**, AdoCbl density in *Ab*Pcob (PDB code: 8J2Y). **e**, AdoCbl and BV in *Sas*Pcob dark state (PDB code: 8J2W). **f.** AdoCbl and BV in *Sas*Pcob light state (PDB code: 8J2X). AdoCbl and BV molecules are shown as magenta and yellow sticks. **g-h.** Crystal packing for dark and light models. Cartoon representation of symmetry elements found in dark (**g**) and light (**h**) structures within 12 Å of the protein backbone atoms. The individual chains present in the asymmetric unit are colored in red and green and the symmetry elements are shown in grey. The individual chains present in the asymmetric unit are colored in red and green and the symmetry elements are shown in grey.

**Supplementary Figure 7. Analysis of *Ab*Pcob and *Ab*DPcob structures after MD simulation. a**, Root Mean Square Deviation (RMSD) of Cα atoms during the simulation time of *Ab*Pcob in light and dark state. **b**, Root Mean Square Fluctuations (RMSF) of Cα atoms during the simulation time of *Ab*Pcob in light and dark state. **c**, Root Mean Square Deviation (RMSD) of Cα atoms during the simulation time of *Ab*DPcob in light and dark state. **d**, Root Mean Square Fluctuations (RMSF) of Cα atoms during the simulation time of *Ab*DPcob in light and dark state. DGC region with conformational change was highlighted by a black circle. **e**, RMSF of residues superposed onto *Ab*DPcob in light and dark states (**f**), with the most (least) variable regions depicted in red (blue).

**Supplementary Figure 8. Additional sampling simulation of *Ab*DPcob in dark and light states. a-b**, Root Mean Square Deviation (RMSD) of Cα atoms during the simulation time of *Ab*DPcob in dark (**a**) and light (**b**) state for different anneal run (a, b, c). **c-d**, Root Mean Square Fluctuations (RMSF) of Cα atoms during the simulation time of *Ab*DPcob in dark (**c**) and light (**d**) state. **e-f**, structure aligment of *Ab*DPcob structure in dark (**e**) and light (**f**) state after sampling simulation. The starting structure was coloured as gray and different anneal run as green (anneal a), blue (anneal b), yellow (anneal c).

**Supplementary Figure 9. BV docking into simulated *Ab*DPcob structure.** 100 clusters were obtained with RMSD cut-off of 1.0 Å following simulated annealing. Represented BV bind poses are shown in the figure. **a**, cluster 23 model 2; **b**, cluster 5 model 2; **c**, cluster 15 model 1. Proteins are shown as grey cartoon. BV and AdoCbl molecules are shown as yellow and magenta sticks, respectively. Residues involved with binding are shown as grey sticks.


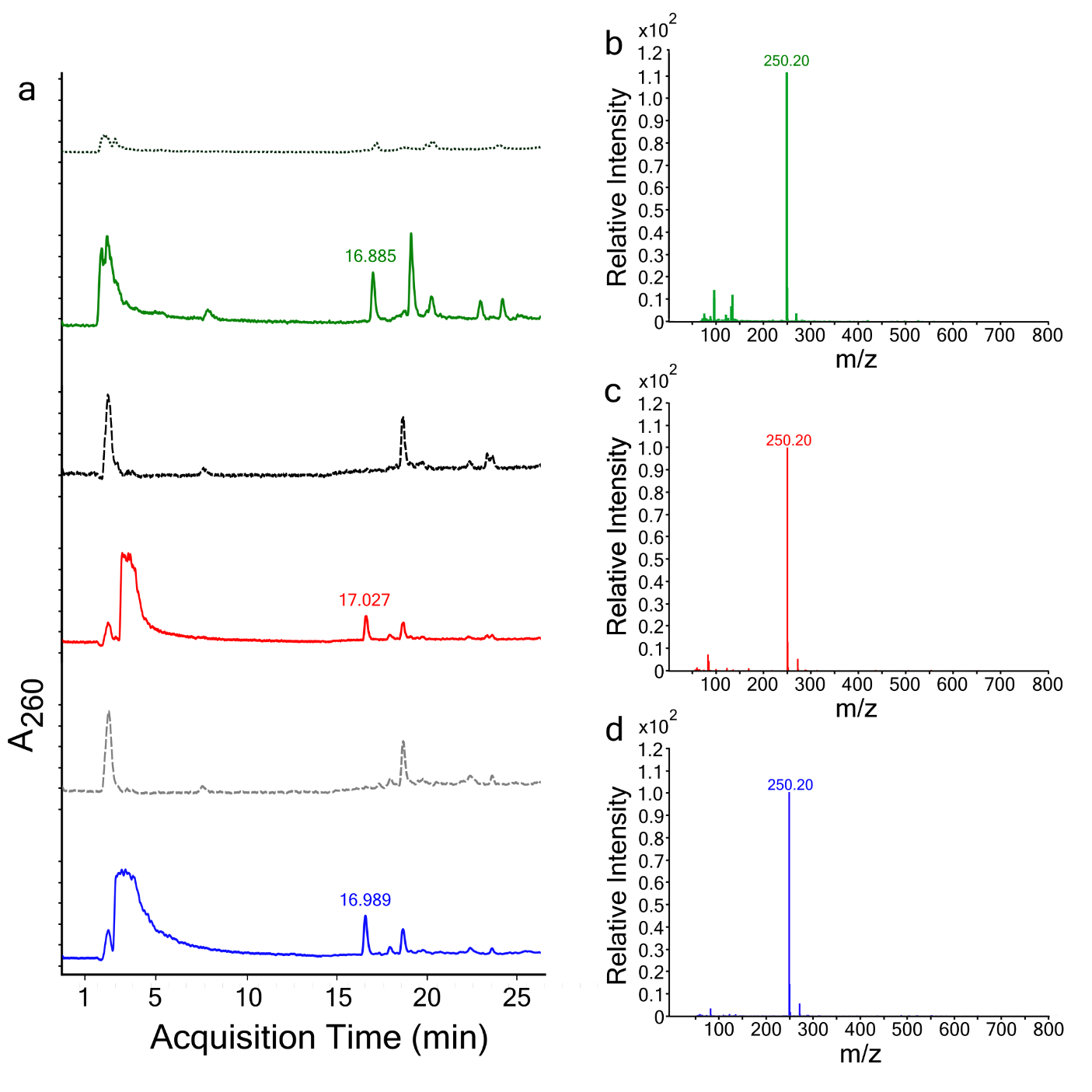


**Supplementary Figure 10. LC-MS analysis to determine the photoproduct of *Sas*Pcob and *Ab*DPcob photoreaction.** Supernatant was collected for dark state and light exposed protein. Panel **a** shows the HPLC traces for CarH (dark green dots), CarH exposed to light (green), *Sas*Pcob (black dashed), *Sas*Pcob exposed to light (red), *Ab*DPcob (grey dashed) and *Ab*DPcob exposed to light (blue). For all light spectra a peak was observed at 17 minutes which corresponded to masses observed at 250.20 (panels **b-d** respectively).


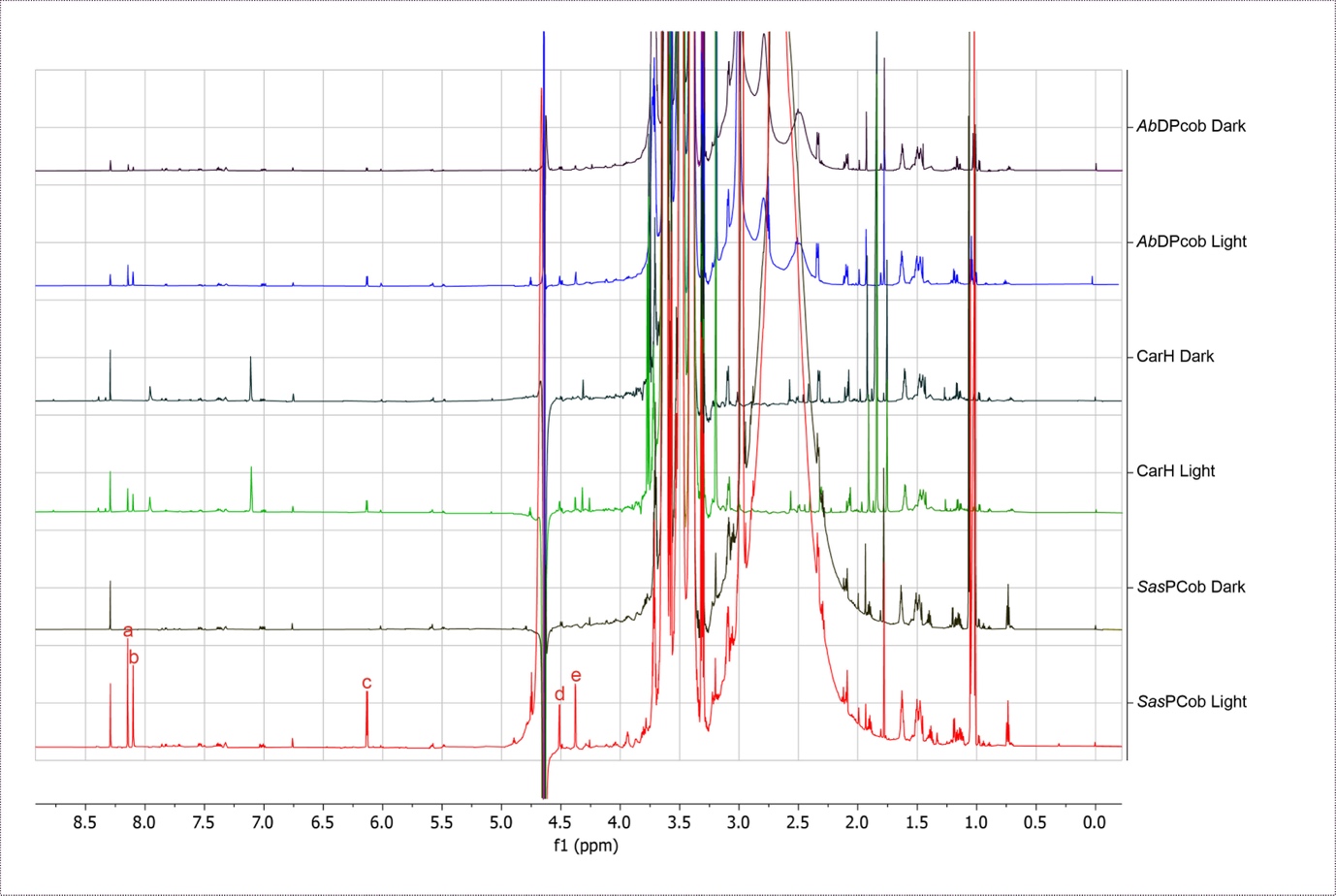


**Supplementary Figure 11**. **1D 1H NMR spectra of CarH, *Sas*PCob and *Ab*DPcob**. Results show the formation of the same product after exposure to light. This compound is comparable to Jost. *et al* 2015 but cannot be fully elucidated due to overlap with residual water signals. Peaks a-e indicate product resonances corresponding to values 8.14, 8.09, 6.13, 4.51 and 4.38 respectively.

**
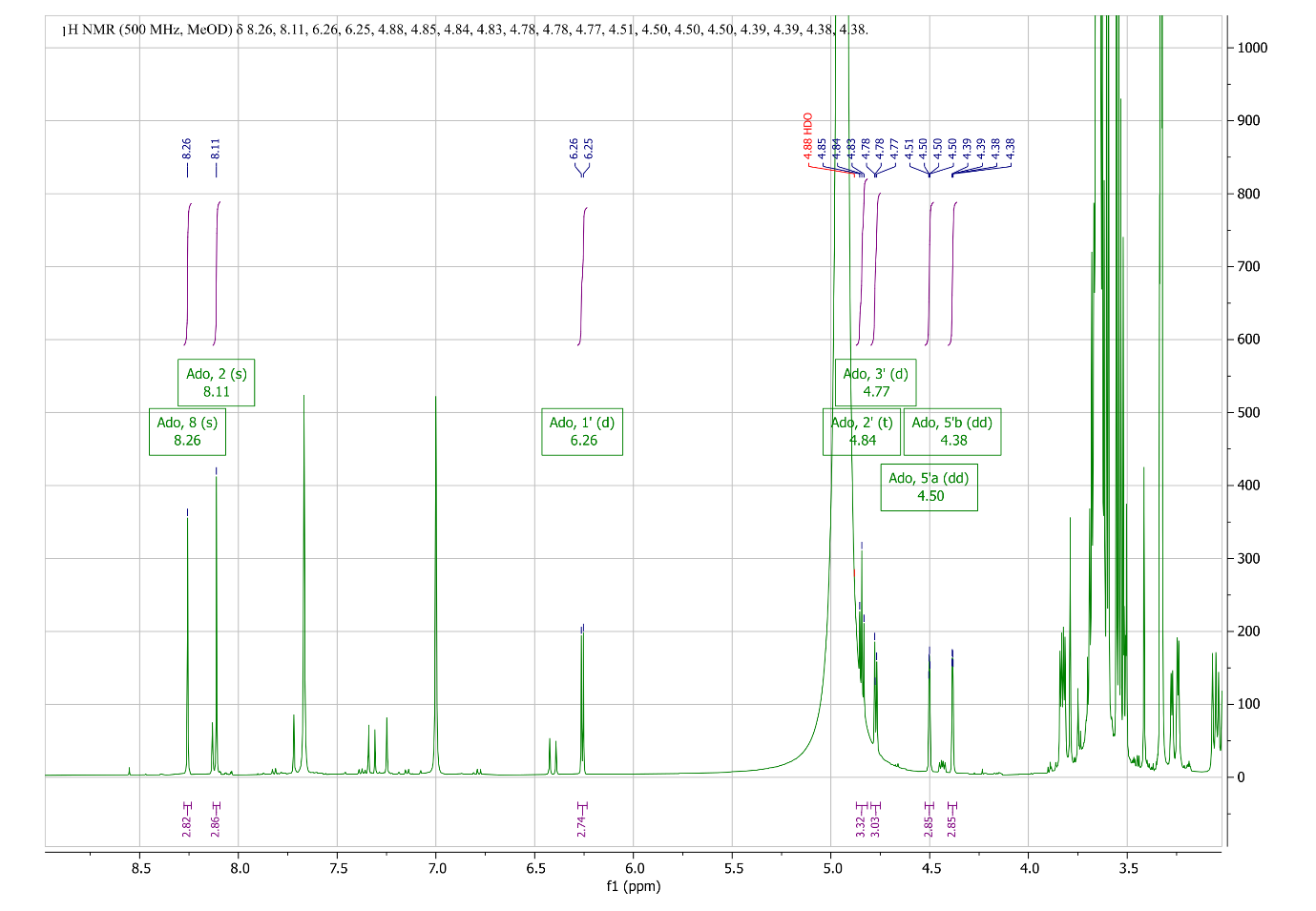
**

**Supplementary Figure 12**. **1D 1H NMR spectrum of illuminated CarH product in deuterated methanol**. Further NMR experiments with higher concentrations of protein show the same product with better peak resolution. This compound (and its numbering) is comparable to Jost. *et al* 2015.


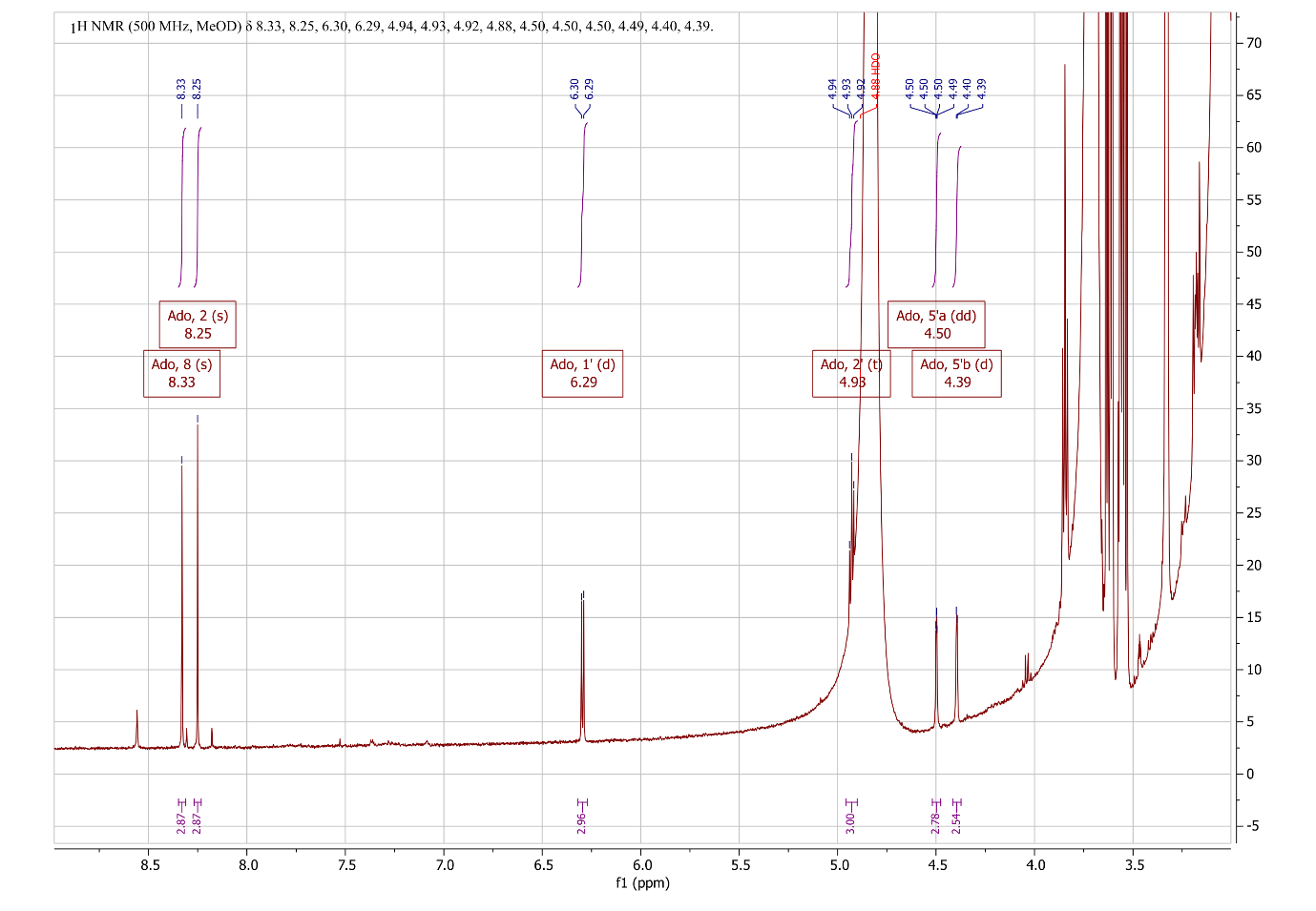


**Supplementary Figure 13**. **1D 1H NMR spectrum of illuminated SasPcob product in deuterated methanol**. Further NMR experiments with higher concentrations of protein show the same product with better peak resolution. This compound (and its numbering) is comparable to Jost. *et al* 2015.

*
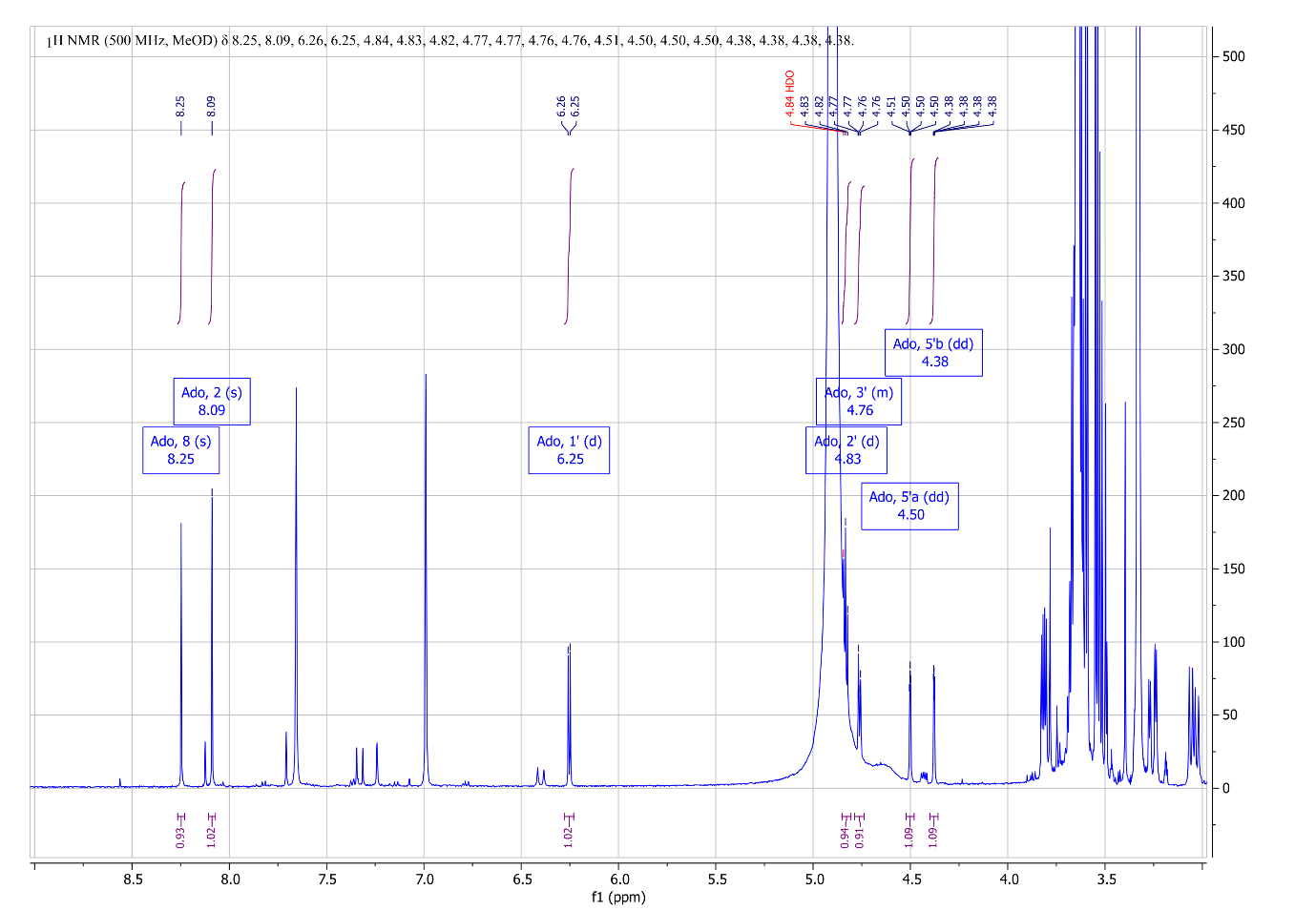
*

**Supplementary Figure 14**. **1D 1H NMR spectrum of illuminated CarH and SasPcob mixture in deuterated methanol**. Slight differences in solvation led to different proton chemical shifts for CarH and SasPcob. To determine whether the compound is the same in both samples another 1D 1H spectrum was generated with the products mixed to produce roughly equivalent concentrations showing only one set of peaks.


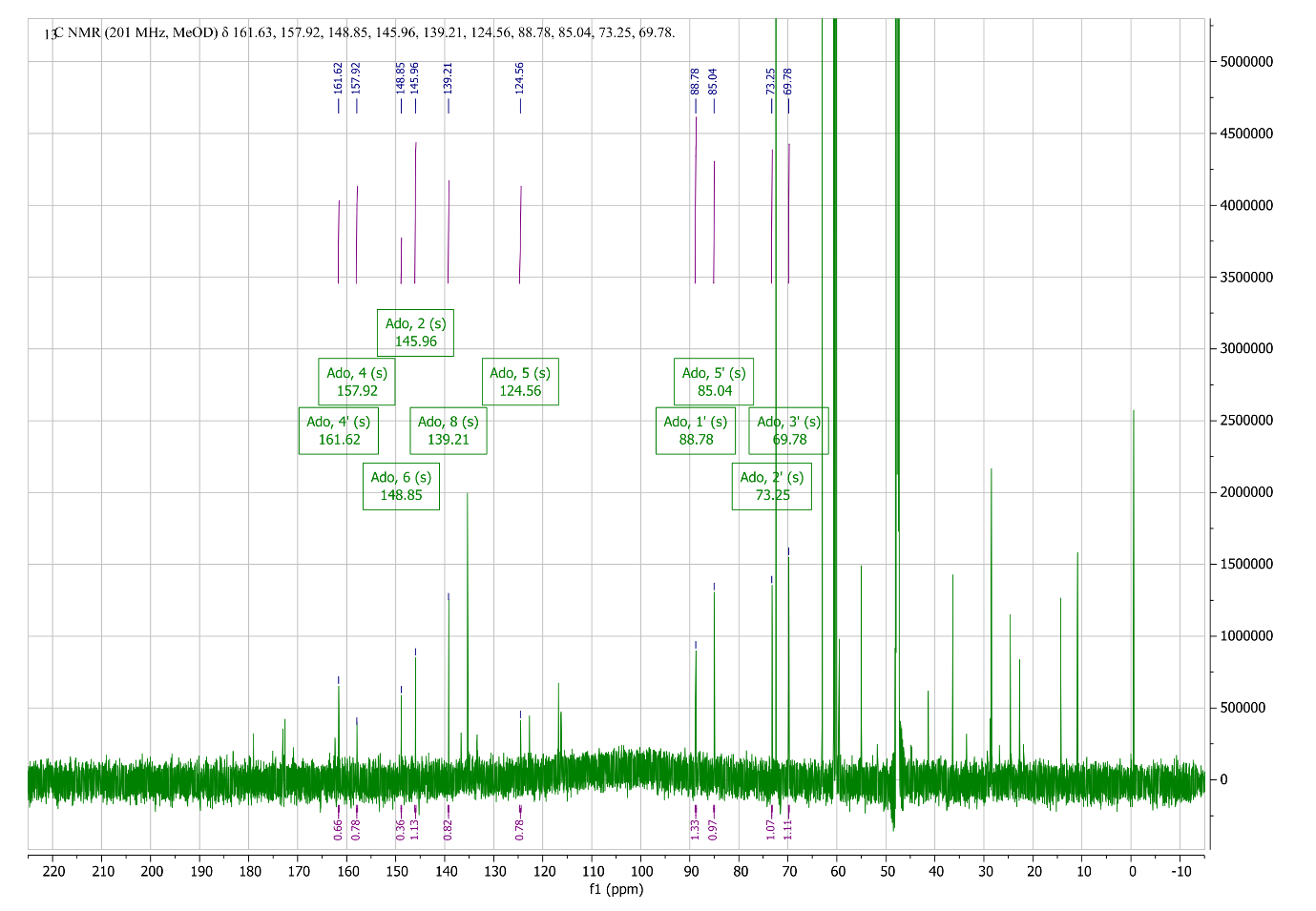


**Supplementary Figure 15**. **1D 13C NMR spectrum of illuminated CarH product in deuterated methanol**. This compound (and its numbering) is comparable to Jost. *et al* 2015.


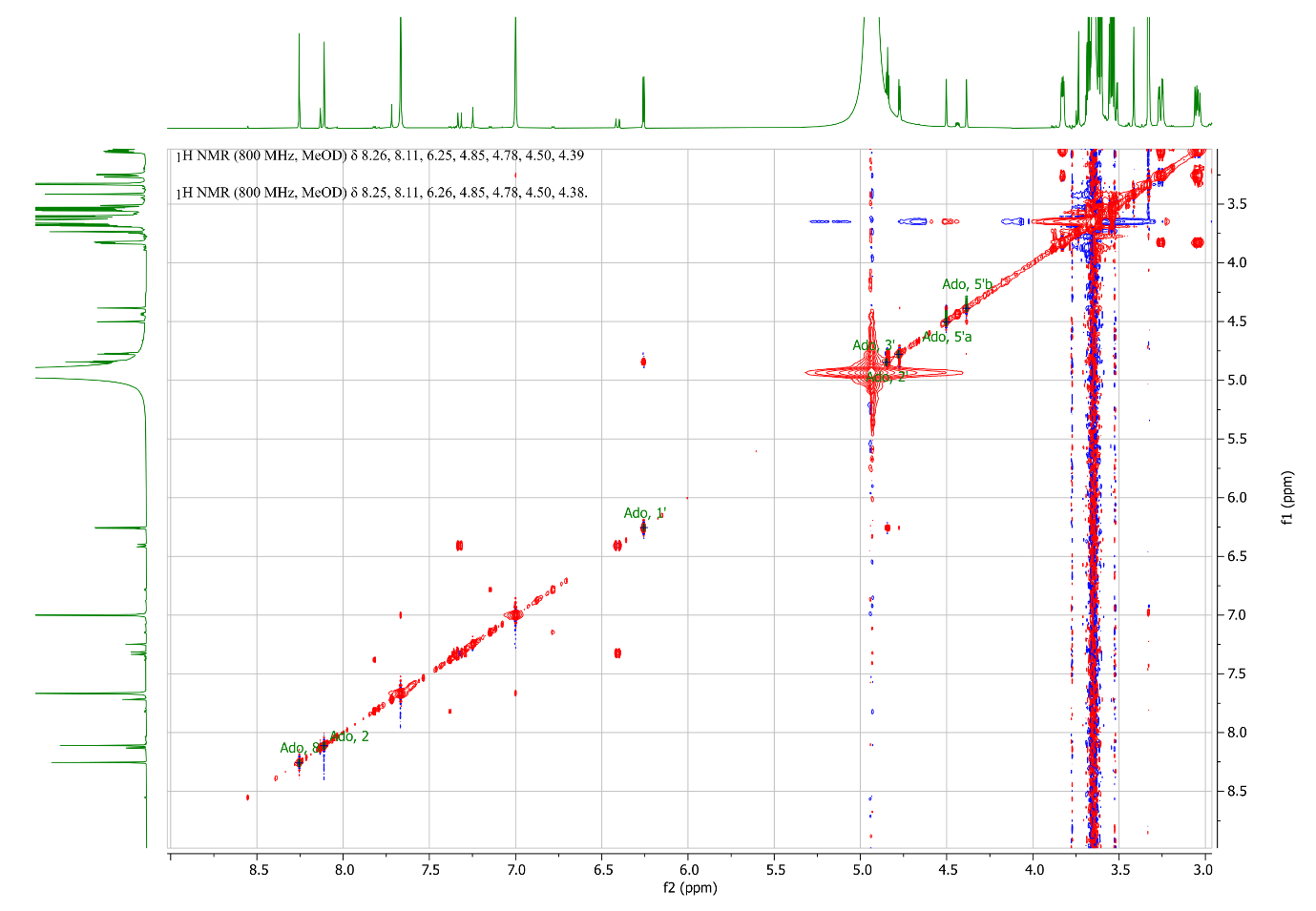


**Supplementary Figure 16**. **CLIP-COSY NMR spectrum of illuminated CarH product in deuterated methanol**. This compound (and its numbering) is comparable to Jost. *et al* 2015.


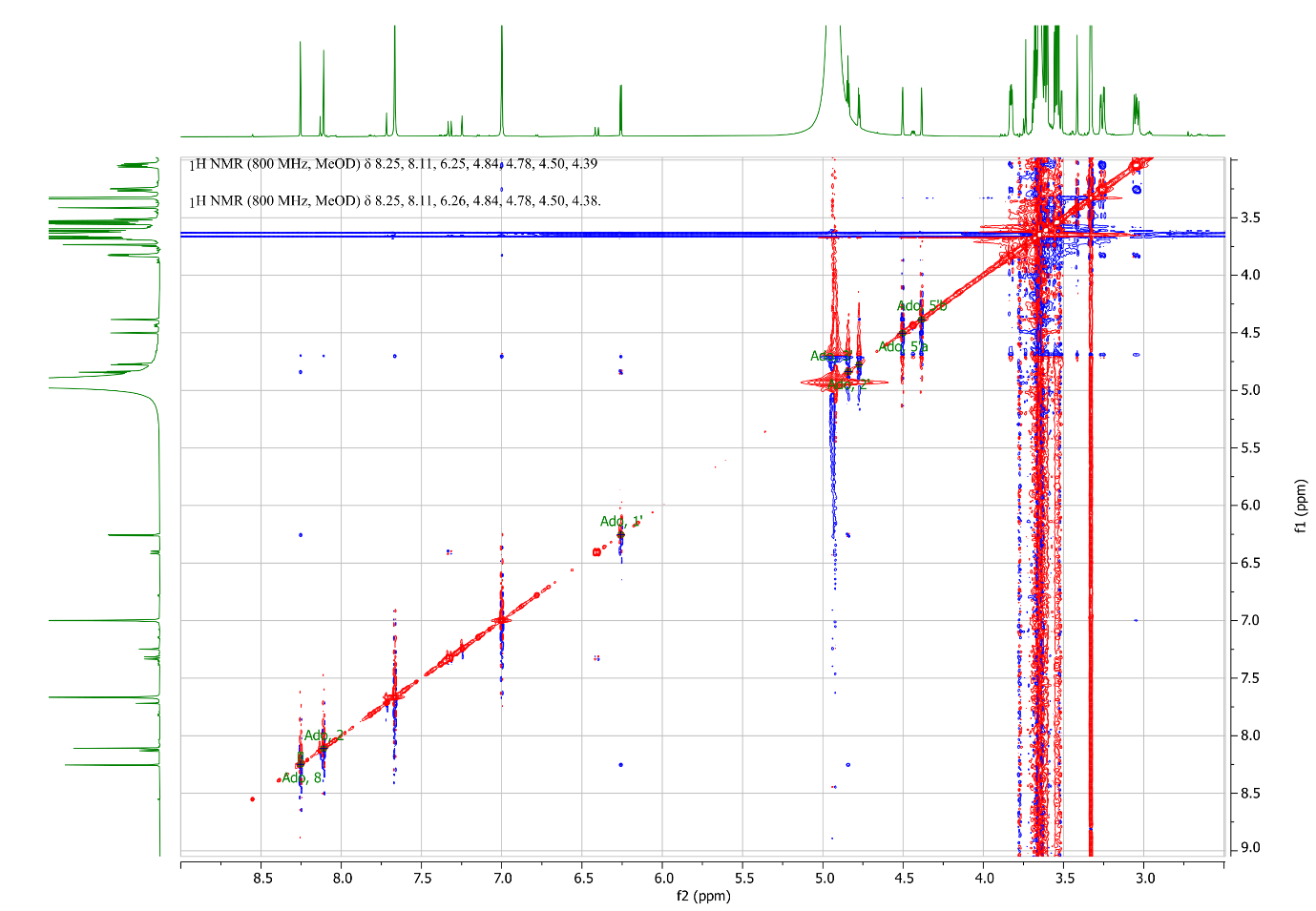


**Supplementary Figure 17**. **EASY-ROESY NMR spectrum of illuminated CarH product in deuterated methanol**. This compound (and its numbering) is comparable to Jost. *et al* 2015.


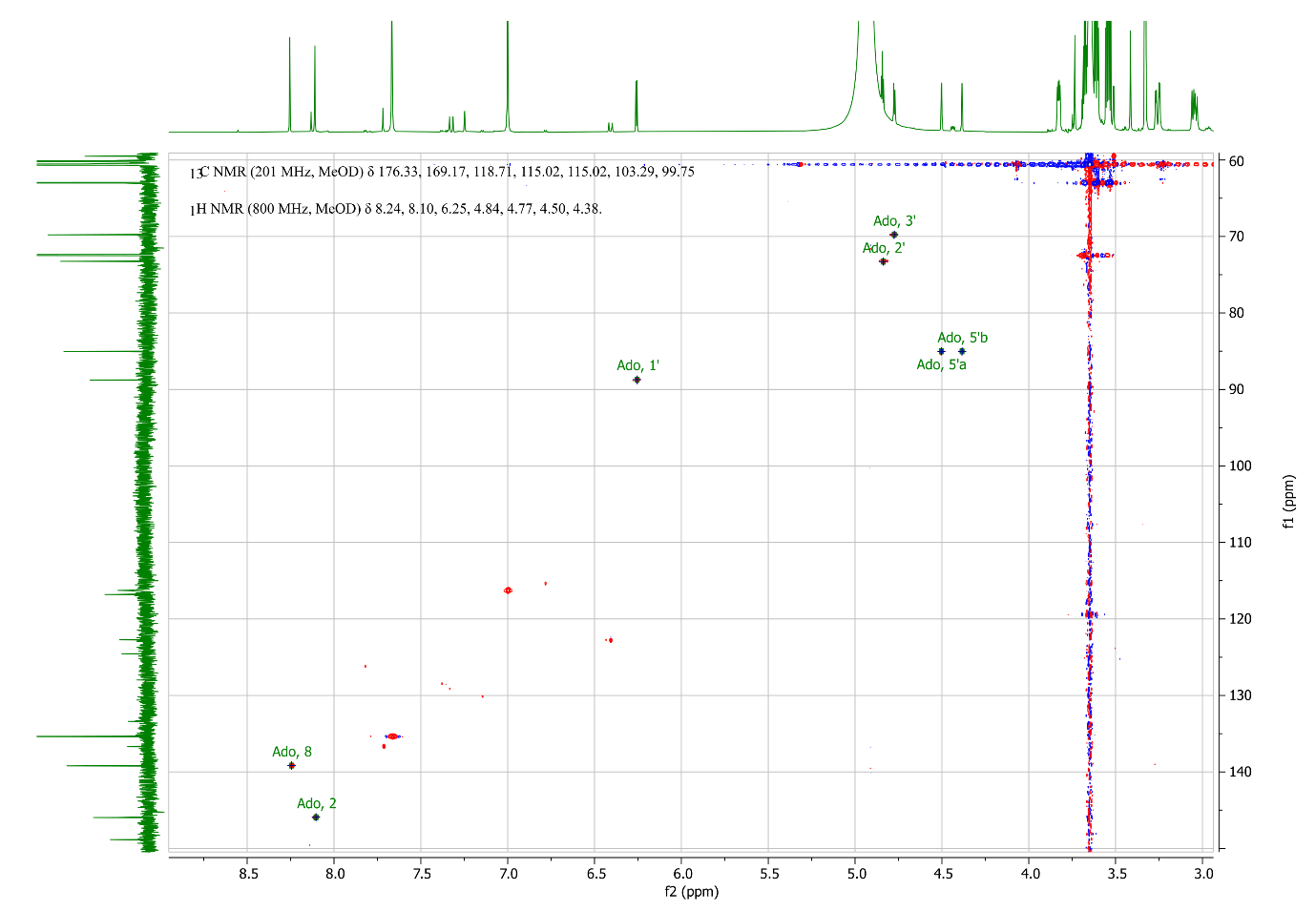


**Supplementary Figure 18**. **HSQC NMR spectrum of illuminated CarH product in deuterated methanol**. This compound (and its numbering) is comparable to Jost. *et al* 2015.


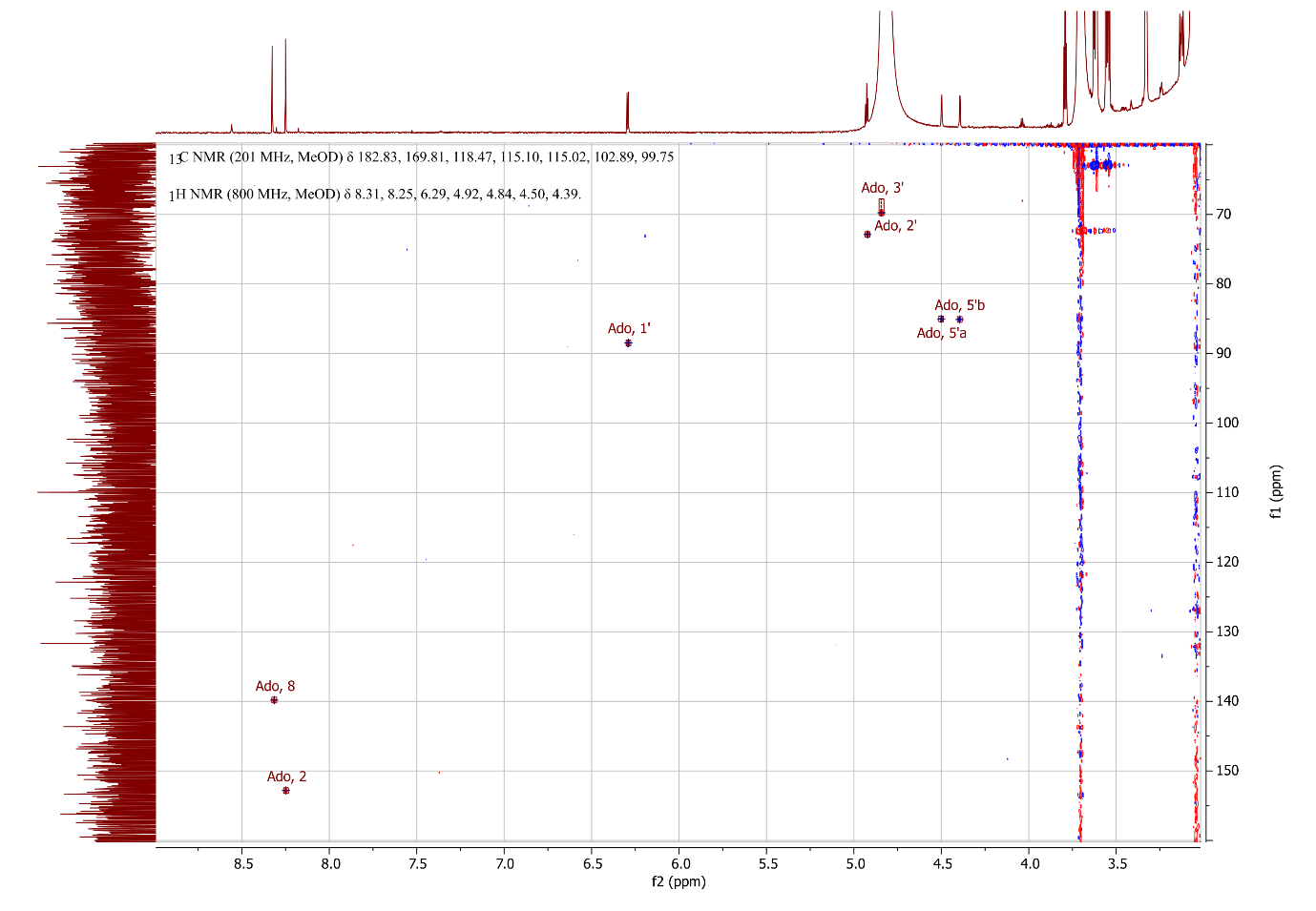


**Supplementary Figure 19**. **HSQC NMR spectrum of illuminated SasPcob product in deuterated methanol**. This compound (and its numbering) is comparable to Jost. *et al* 2015.


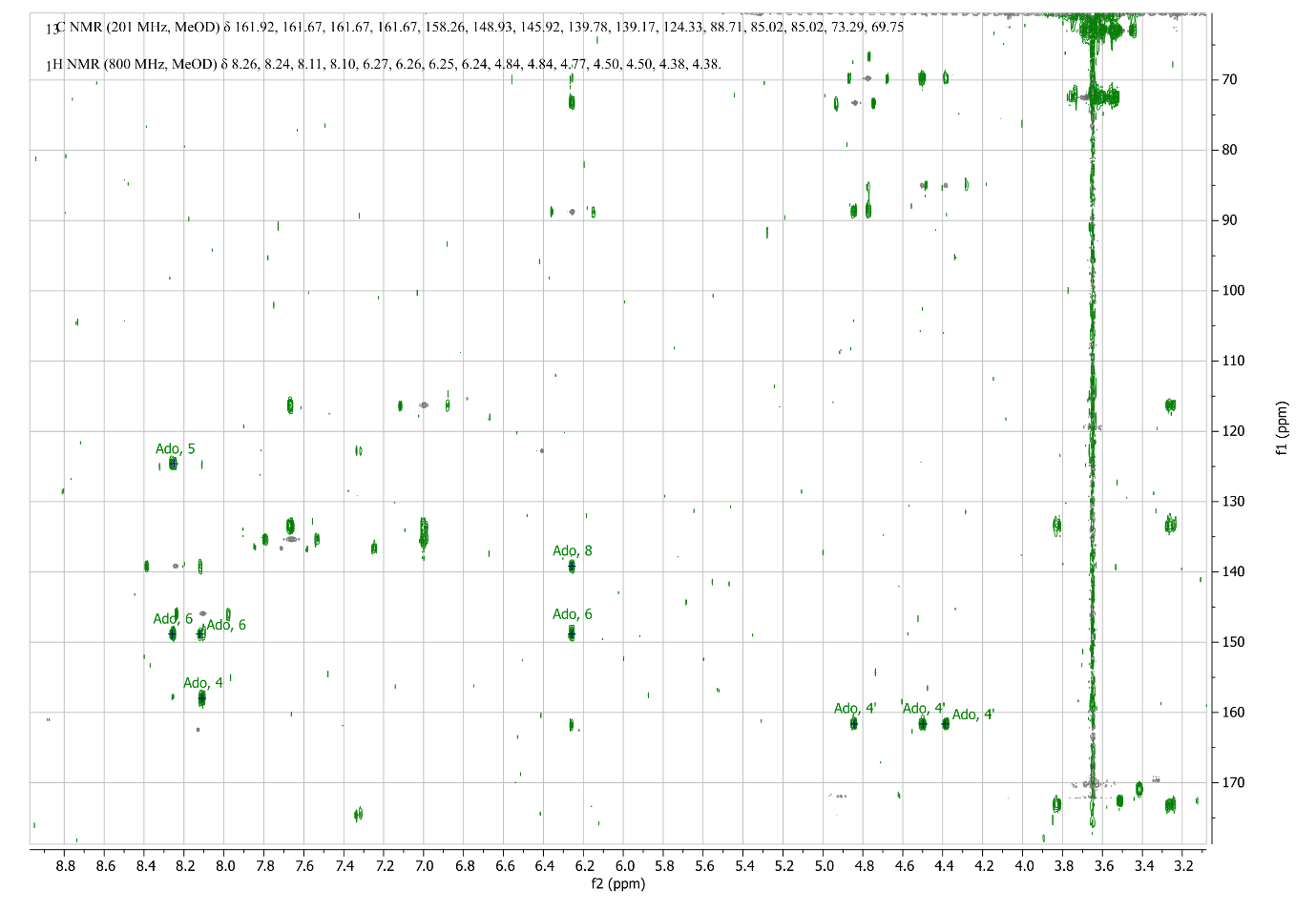


**Supplementary Figure 20**. **HMBC NMR spectrum overlaid with HSQC NMR spectrum of illuminated CarH product in deuterated methanol**. The HMBC is shown in purple and the HSQC in grayscale. This compound (and its numbering) is comparable to Jost. *et al* 2015.


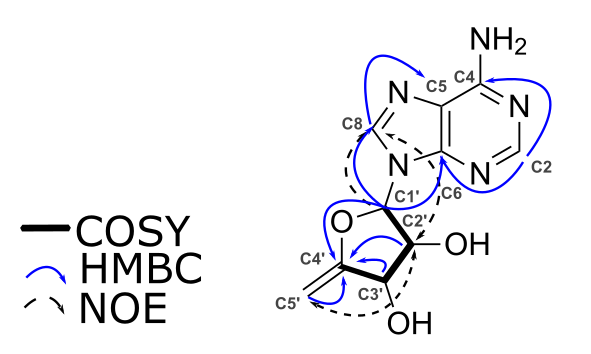


**Supplementary Figure 21**. **Schematic of the intramolecular interactions of 4’,5′-anhydroadenosine**


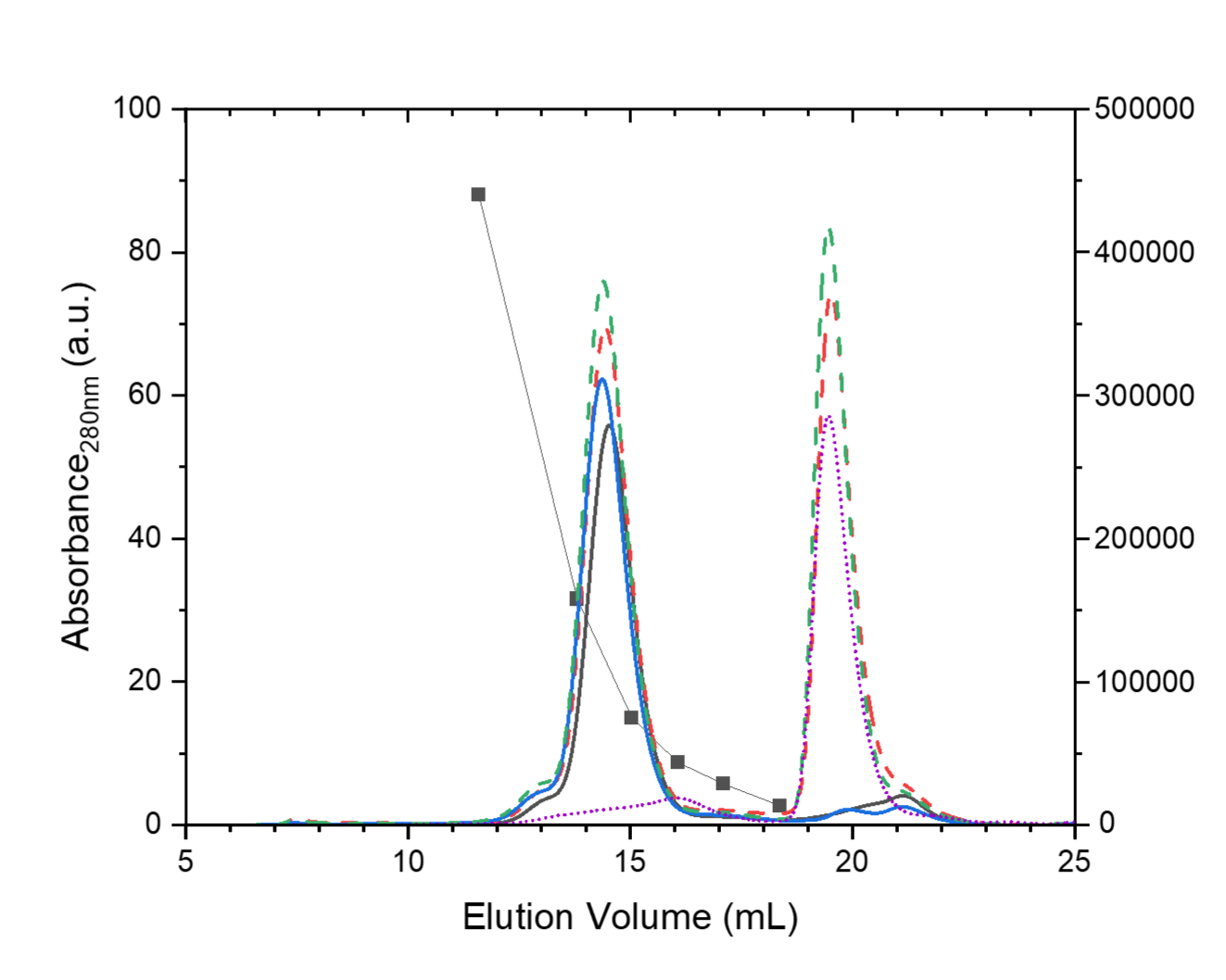


**Supplementary Figure 22. Full-length *Ab*DPcob protein was analysed on an analytical gel filtration column to determine mass and oligomerisation states.** Curves correspond to full-length dimeric protein showed no change in oligomerization. *Ab*DPcob without GTP (solid lines) showed a single peak at around 14-15 mL whether in the dark (black solid) or light-exposed states (blue solid). When *Ab*DPcob was present two peaks were observed (dashed lines) corresponding to the full-length protein at 14-15 mL and unbound GTP at 19-20 mL whether in the dark (red dashed) or light-exposed states (green dashed). All protein peaks corresponded to the dimeric full-length protein. GTP was also ran with no protein present producing a single peak at 19-20 mL (purple dotted line). The calibration curve for masses observed is shown in black squares.


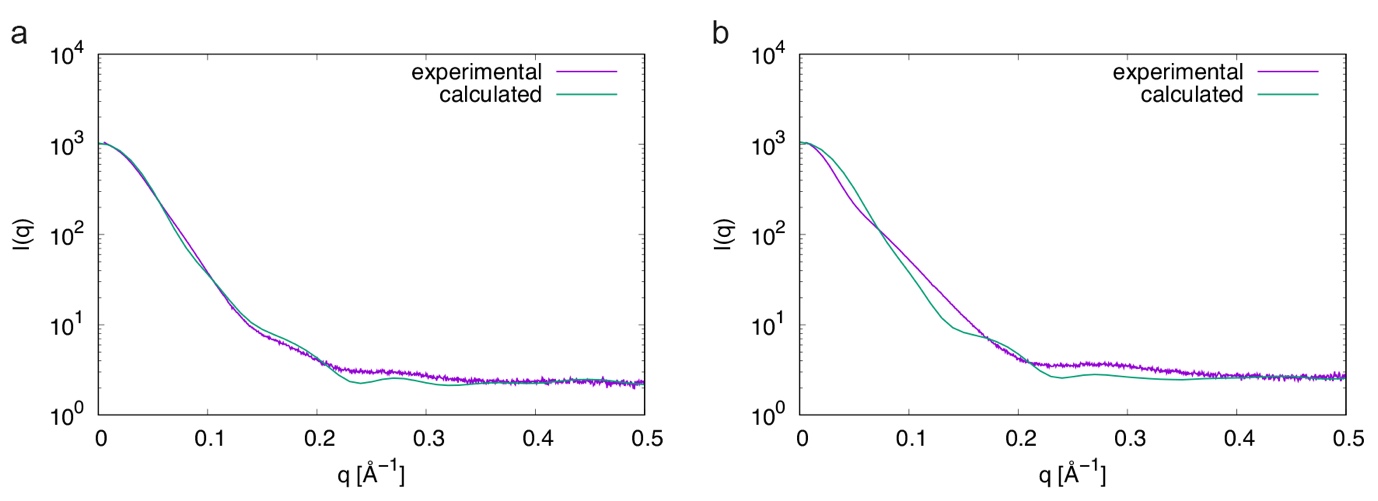


**Supplementary Figure 23. Comparison of calculated theoretical curve with *Ab*DPcob SAXS experimental data.** Theoretical curves calculated with AlphaFold2 *Ab*DPcob dimer model. *Ab*DPcob contain AdoCbl as dark state (**a**) and *Ab*DPcob with OHCbl after MD simulation as light state (**b**). Experimental and calculated curves are shown as purple and green respectively. Comparison of dark state reveal overall but not full agreement. The theoretical curves were calculated using the molecular dynamics simulation based server WAXSIS ([https://waxsis.uni-saarland.de/ [waxsis.uni-saarland.de]](https://urldefense.com/v3/__https:/waxsis.uni-saarland.de/__;!!PDiH4ENfjr2_Jw!HOUH-4RyQqAjyeMtIFOrHo5djPT8aoFqHcTJkh4-TKvdh3SWevn66ZtN_Uoo0Fndi-HOBUFJW7ElBtCbg4-my2qRvAcXI1Kaaw$)) using the pdb files produced by AlphaFold2. Agreement of light state is very poor, which might due to the limitation of MD simulation. To give a real light state conformation, further MD simulation is needed.

**Supplementary Figure 24. The protein-protein interaction network analysis showing the functions of potential partner proteins for standalone photocobilins. a**, The potential protein-protein interaction network of proteins for four species. Each node represents a protein with mapped proteins of 506 sequences in a certain species coloured in a darker colour, while predicted interacting proteins are coloured in a light colour. The edges between nodes represent the confidence score that integrates the protein-protein interaction information, containing text-mining, co-expression, gene neighbourhood, gene fusion, co-occurrence, etc. The intensity of the colour represents a more confident interaction between two proteins. **b**, TEnrichment analysis based on gene list from protein-protein interaction network in different species. Terms with detailed descriptions are annotated in the figure.

**Supplementary Figure 25. Bioinformatic and structural analysis of photocobilin-containing proteins. a,** PPI network analysis of Pcob proteins. The functional enrichment analysis of potential protein-protein interaction network predicted by the STRING database^40^ in four species. Enriched terms (p-value < 0.05) that occurs in at least two species are shown. **b-d**, Structural alignment of predicted AlphaFold2 structures with *Sas*Pcob. Protein structures are shown as cartoons. B_12_ and BV domain of *Sas*Pcob are coloured as blue and orange respectively. Predicted structures and extra functional domains are shown as green and magenta cartoons.

**
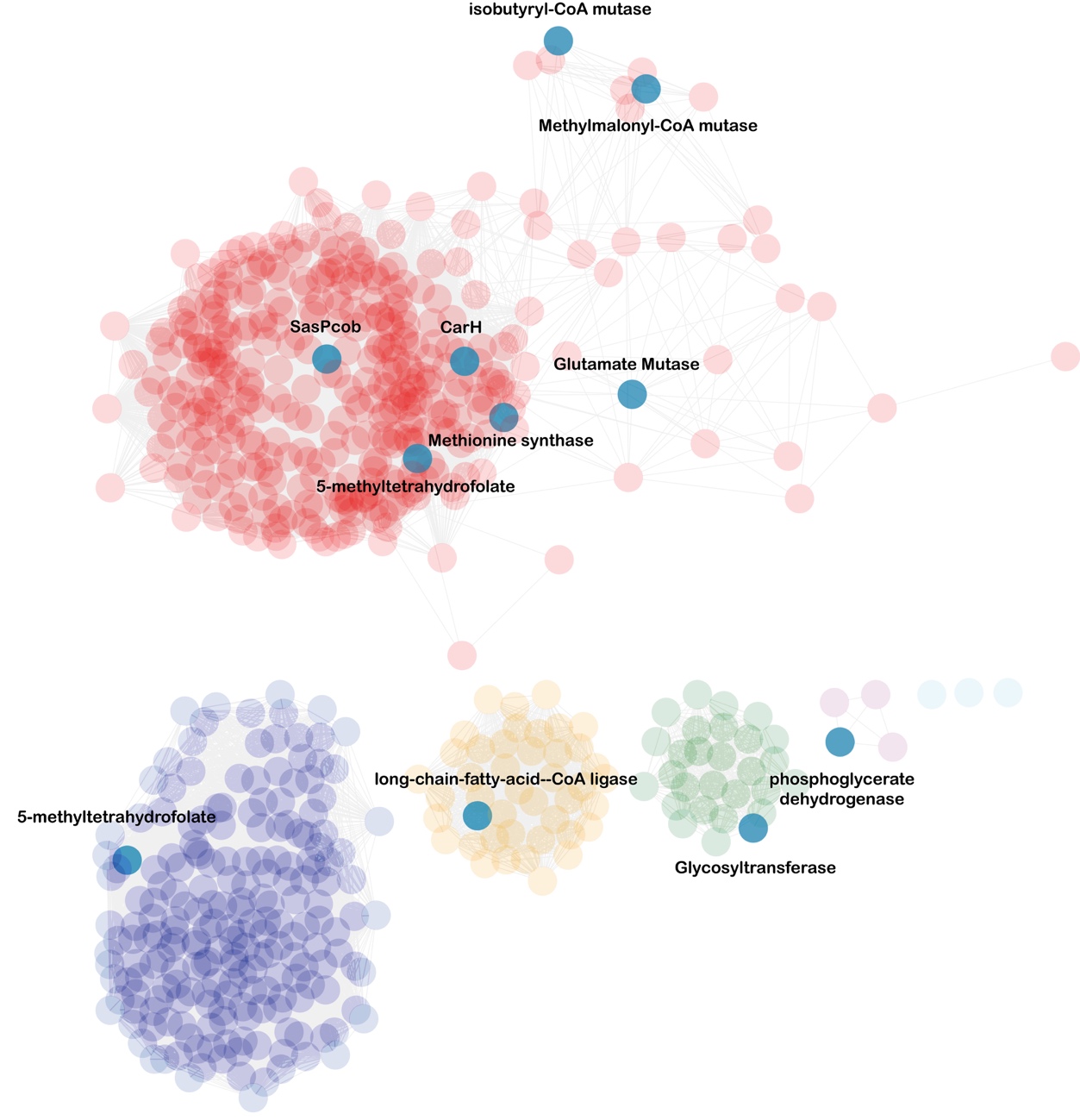
**

**Supplementary Figure 26. A clustering analysis of the SasPcob-related sequence space.** A total of 648 UniRef40 sequence, identified through BLASTp analysis, were used for further analysis. The network was visualized using Cytoscape with a force-directed layout with key nodes manually annotated to represent various protein clusters.

**
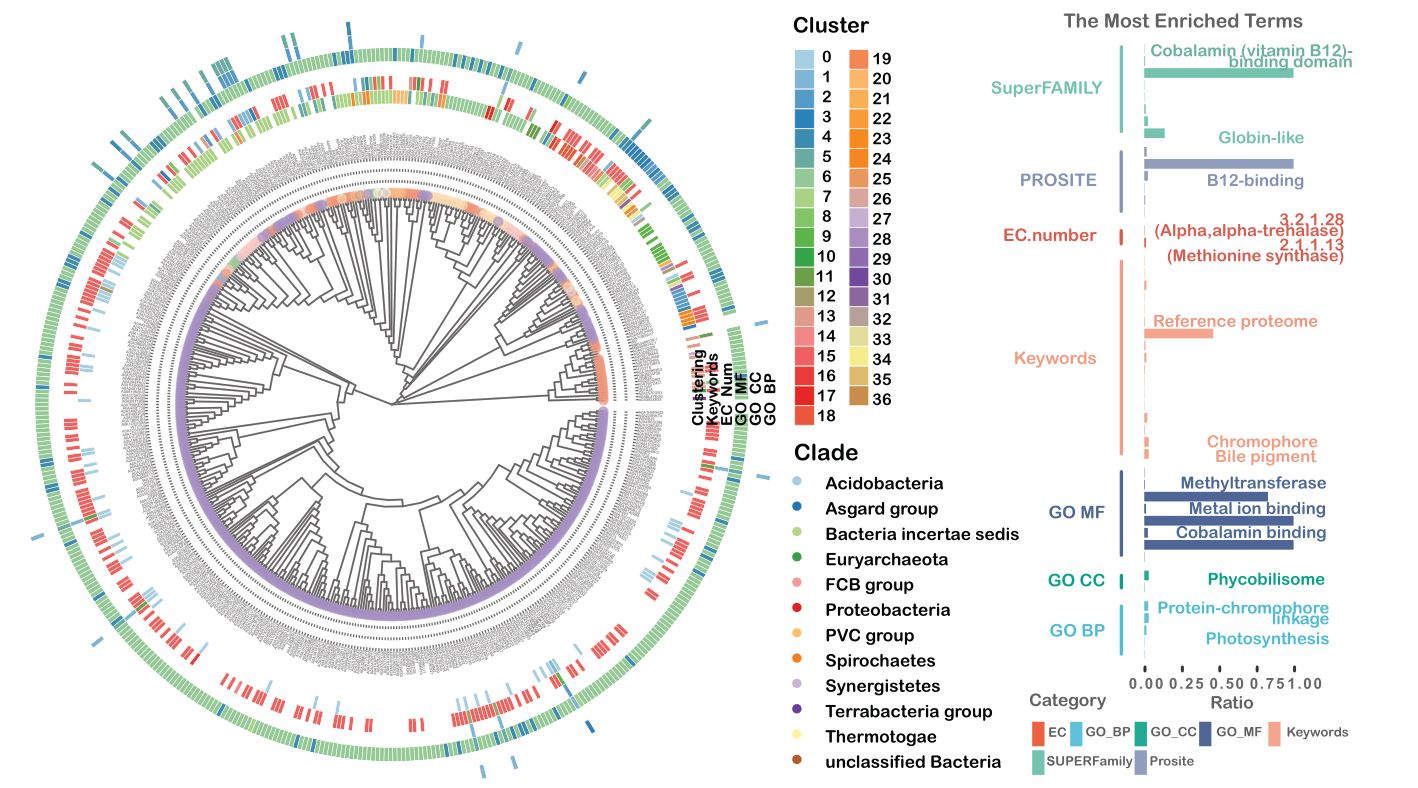
**

**Supplementary Figure 27. Phylogenetic analysis of photocobilin-containing proteins. a**, Phylogenetic analysis and functional annotation of 506 photocobilin -containing proteins. 506 sequences were grouped into 37 clusters using three rounds of cd-hit software. Barplot showing the summary of functional annotations.


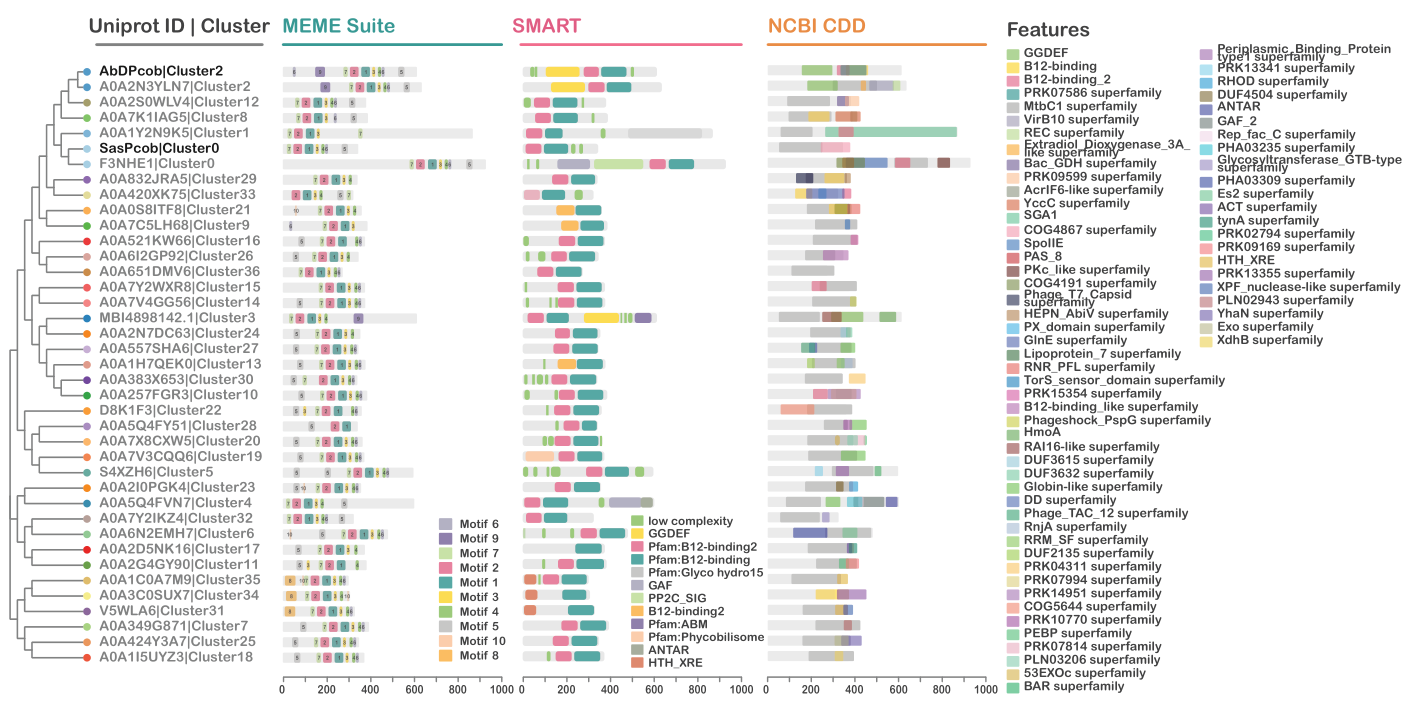


**Supplementary Figure 28. Feature annotation of photocobilin-containing proteins.** Motif and domain prediction of representative protein sequence in 37 clusters. Motif predictions were performed using MEME Suite, Domain predictions were performed based on SMART and NCBI CDD tools.

**
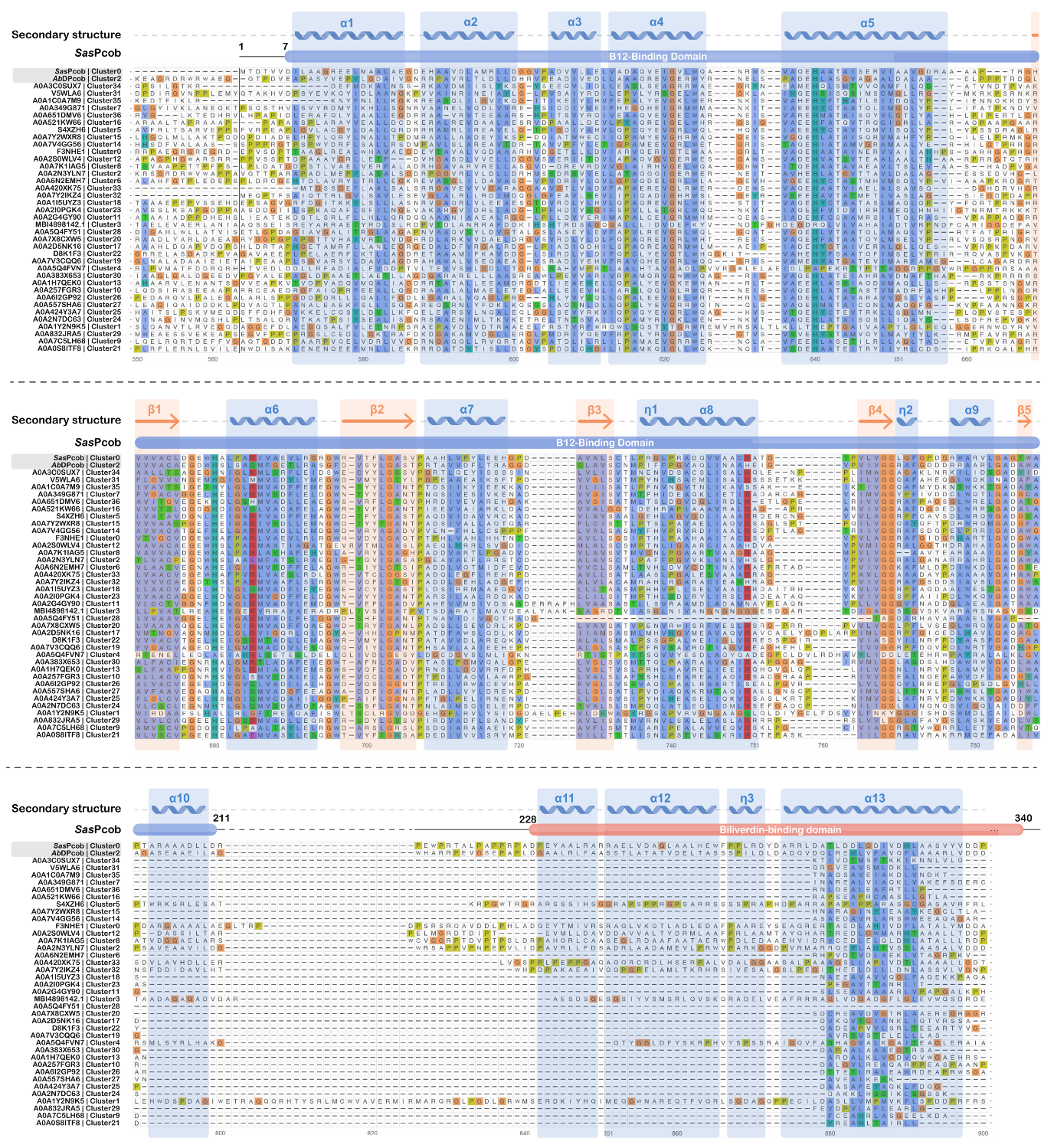
**

**Supplementary Figure 29. Multiple sequence alignment and structural annotation of the representative sequence of 37 clusters.** The sequence of *Sas*Pcob and *Ab*DPcob are included in the analysis, which also shows secondary structure information and motif prediction.

**Supplementary Figure 30. Representative domains located at photocobilin-containing gene loci. a,** Genome neighbourhood distribution of photocobilins used in this study. *Sas*Pcob and *Ab*DPcob are shown as red box and other genes are named and coloured by their domains. **b,** Selected representative photocobilins and their gene loci. Each gene is represented as a box. The photocobilin is shown as the red box, while other boxes represent the Pfam families as indicated. The direction of the box shows the direction of the gene transcription. Apart from photocobilin, intensity of the colour shade represents the gene frequency of occurrence in the position.

**Supplementary Figure 31. Comparison of predicted photocobilin structures and sequences with *Sas*Pcob.** Representative photocobilins were selected, grouped according to domain organisation and Alphafold2 models downloaded^1, 2^. **a-d**, Structure alignment of predicted Alphafold2 structures with *Sas*Pcob. Protein structures are shown as cartoons. B_12_ and BV domain of *Sas*Pcob are coloured blue and orange respectively. Predicted structures and extra functional domains are shown as green and magenta cartoons. **e,** Multiple sequence alignment of selected photocobilins with *Sas*Pcob. The alignment result is coloured according to sequence identity by MView^3^.

**Supplementary Tables:**

**Supplementary Table 1.** Structures distribution after clustering of *Ab*Pcob and their maximum binding affinity to BV.

| Cluster No. | Structures | Max binding affinity |
| --- | --- | --- |
| 1 | 1734 | >0 |
| 2 | 1262 | >0 |
| 3 | 779 | >0 |
| 4 | 232 | -5.5 |
| 5 | 228 | -0.8 |
| 6 | 159 | -7.9 |
| 7 | 132 | >0 |
| 8 | 84 | -6.1 |
| 9 | 78 | -6.4 |
| 10 | 72 | -6.4 |
| 11 | 47 | -2.7 |
| 12 | 29 | -9.7 |
| 13 | 26 | >0 |
| 14 | 19 | -1.6 |
| 15 | 15 | >0 |
| 16 | 13 | -4.9 |
| 17 | 13 | -2 |
| 18 | 12 | -2.1 |
| 19 | 11 | -8.4 |
| 20 | 7 | -6.7 |
| 21 | 7 | -0.9 |
| 22 | 7 | >0 |
| 23 | 4 | -6.2 |
| 24 | 4 | -4.2 |
| 25 | 4 | >0 |
| 26 | 3 | -6.9 |
| 27 | 3 | -4.9 |
| 28 | 3 | -4.8 |
| 29 | 2 | >0 |
| 30 | 2 | >0 |
| 31 | 2 | -5.1 |
| 32 | 2 | >0 |
| 33 | 1 | -5.2 |
| 34 | 1 | -3.2 |
| 35 | 1 | -7.6 |
| 36 | 1 | -3.1 |
| 37 | 1 | -1.7 |
| 38 | 1 | >0 |

**Supplementary Table 2.** Structures distribution after clustering of *AbD*Pcob from MD simulations following annealing, and their maximum binding affinity to BV.

| Cluster No. | BV site^1^ | Structures | Max binding affinity |
| --- | --- | --- | --- |
| 1 | 1 | 6790 | -8 |
| 2 | 1 | 3503 | -5.6 |
| 3 | 2 | 2482 | -8.7 |
| 4 | 2 | 2478 | -7.8 |
| 5 | 2 | 2416 | -8.6 |
| 6 | 2 | 739 | -7.6 |
| 7 | 2 | 615 | -4.6 |
| 8 | 2 | 447 | -7.9 |
| 9 | 2 | 349 | -7.6 |
| 10 | 2 | 283 | -7.5 |
| 11 | 2 | 213 | -8.6 |
| 12 | 2 | 210 | -7.6 |
| 13 | 1 | 182 | -8.2 |
| 14 | 2 | 82 | -5.6 |
| 15 | 1 | 35 | -8.8 |
| 16 | 2 | 33 | -8.5 |
| 17 | 2 | 20 | -8.5 |
| 18 | 2 | 19 | 0.1 |
| 19 | 2 | 16 | -8 |
| 20 | 2 | 10 | -8.5 |
| 21 | 2 | 8 | -8.2 |
| 22 | 2 | 7 | -6.2 |
| 23 | 2 | 5 | 0 |
| 24 | 2 | 3 | -6.7 |
| 25 | 2 | 3 | -7.9 |
| 26 | 2 | 3 | -9.7 |
| 27 | 2 | 3 | -8.3 |
| 28 | 2 | 3 | -7.9 |
| 29 | 2 | 3 | -6.5 |
| 30 | 2 | 3 | -8.7 |
| 31 | 2 | 2 | -7.9 |
| 32 | 2 | 2 | -8.5 |
| 33 | 2 | 2 | -8.7 |
| 34 | 2 | 2 | -8.5 |
| 35 | 2 | 2 | -7.9 |
| 36 | 2 | 2 | -8.3 |
| 37 | 2 | 2 | -7.4 |
| 38 | 2 | 2 | -8.4 |
| 39 | 2 | 2 | -8.2 |
| 40 | 1 | 1 | -7.1 |
| 41 | 1 | 1 | -7.9 |
| 42 | 1 | 1 | -3.6 |
| 43 | 1 | 1 | -8 |
| 44 | 1 | 1 | -8.4 |
| 45 | 1 | 1 | -7.2 |
| 46 | 1 | 1 | -8.6 |
| 47 | 1 | 1 | -8 |
| 48 | 1 | 1 | -8.8 |
| 49 | 1 | 1 | -9.1 |
| 50 | 1 | 1 | -7.6 |
| 51 | 2 | 1 | -8.9 |
| 52 | 2 | 1 | -8 |
| 53 | 2 | 1 | -8.1 |
| 54 | 2 | 1 | -8.3 |
| 55 | 2 | 1 | -9 |
| 56 | 2 | 1 | -8.7 |
| 57 | 2 | 1 | -7.8 |
| 58 | 2 | 1 | -8.1 |
| 59 | 2 | 1 | -8.9 |
| 60 | 2 | 1 | -8.3 |
| 61 | 2 | 1 | -7.8 |
| 62 | 2 | 1 | -8.8 |
| 63 | 2 | 1 | -8.7 |
| 64 | 2 | 1 | -8.9 |
| 65 | 2 | 1 | -8.7 |
| 66 | 2 | 1 | -8.2 |
| 67 | 2 | 1 | -8.2 |
| 68 | 2 | 1 | -8 |
| 69 | 2 | 1 | -10 |
| 70 | 2 | 1 | -10 |
| 71 | 2 | 1 | 0 |
| 72 | 2 | 1 | -4.6 |
| 73 | 2 | 1 | -6.3 |
| 74 | 2 | 1 | -7.6 |
| 75 | 2 | 1 | -8.8 |
| 76 | 2 | 1 | -7.2 |
| 77 | 2 | 1 | -7.8 |
| 78 | 2 | 1 | -8.4 |
| 79 | 2 | 1 | -9.4 |
| 80 | 2 | 1 | -8.5 |
| 81 | 2 | 1 | -8.4 |
| 82 | 2 | 1 | -8.6 |
| 83 | 2 | 1 | -8.7 |
| 84 | 2 | 1 | -9.3 |
| 85 | 2 | 1 | -5.1 |
| 86 | 2 | 1 | -10.3 |
| 87 | 2 | 1 | -9.1 |
| 88 | 2 | 1 | -8.2 |
| 89 | 2 | 1 | -8.1 |
| 90 | 2 | 1 | -9.1 |
| 91 | 2 | 1 | -7.8 |
| 92 | 2 | 1 | -8.3 |
| 93 | 2 | 1 | -7.5 |
| 94 | 2 | 1 | -9.3 |
| 95 | 2 | 1 | -7.9 |
| 96 | 2 | 1 | -9.1 |
| 97 | 2 | 1 | -7.6 |
| 98 | 2 | 1 | -7.4 |
| 99 | 2 | 1 | -8.8 |
| 100 | 2 | 1 | -6.1 |

^1^ Since there are two active sites in *AbD*Pcob clustering and subsequent docking was performed on both.

**Supplementary Table 3.** Data collection, processing and structural parameters of SAXS measurements.

| **SAXS data collection parameters** | | |
| --- | --- | --- |
| Instrument | | BM29 (ESRF, Grenoble) |
| Detector | | Pilatus3 2M |
| Sample-detector distance | | 2.867 m |
| Wavelength | | 0.099187 |
| Beam size | | 200 µm x 200 µm |
| q range | | 0.0025 - 0.5 Å^-1^ |
| Absolute scaling method | | Comparison with pure H_2_O scattering |
| Exposure time | | 990 ms |
| Number of scans | | 10 |
| Sample temperature | | 20 °C |
| **SAXS data reduction parameters** | | |
| Azimuthal integration | | PyFAI |
| Data averaging and buffer subtraction | | Primus-ATSAS 2.8.2 |
| *Ab initio* modeling | | DAMMIF |
| 3D graphic representation | | Chimera |
| **Structural parameters** | | |
|  | **Dark-adapted AbDPcob** | **Light-adapted AbDPcob** |
| Concentration | 5 mg/ml | 5 mg/ml |
| **Guinier analysis** |  |  |
| I(0) (cm^-1^) | 1.03×10³ | 1.08×10³ |
| Rg (Å) | 42.1 | 52.2 |
| Guinier region (Å) | 0.0169 - 0.0313 | 0.0133 - 0.0256 |
| **P(r) analysis** |  |  |
| Rg (Å) | 42.2 | 52.3 |
| d_max_ | 188.16 | 298.0 |
| q range (Å) | 0.0086 - 0.364 | 0.0086 - 0.364 |

**Supplementary Table 4**. DGC activity of *Ab*DGC mixed with different Pcobs domain.

| *Ab*DGC and Pcobs mix with 1:1 ratio | | | | | | | | | |
| --- | --- | --- | --- | --- | --- | --- | --- | --- | --- |
| c-di-GMP produced (mM/min) | AbDGC_only | *Ab*DGC vs *Ab*Pcob dark | *Ab*DGC vs *Ab*Pcob light | *Ab*DGC vs AdoCbl dark | *Ab*DGC vs AdoCbl light | *Ab*DGC vs *Sas*Pcob (bind with B_12_) dark | *Ab*DGC vs *Sas*Pcob (bind with B_12_) light | *Ab*DGC vs *Sas*Pcob (bind with B_12_ and BV) dark | *Ab*DGC vs *Sas*Pcob (bind with B_12_ and BV) light |
| DGC activity 1 | 0.89 | 1.02 | 2.44 | 1.33 | 1.16 | 1.56 | 0.90 | 0.55 | 1.08 |
| DGC activity 2 | 1.05 | 0.94 | 2.47 | 1.17 | 0.96 | 1.60 | 0.56 | 0.56 | 0.86 |
| DGC activity 3 | 0.91 | 0.92 | 2.48 | 1.12 | 1.08 | 1.61 | 0.66 | 0.66 | 1.22 |

**Supplementary Table 5**. DGC activity of *Ab*DGC mixed with *Ab*Pcob domain at different ratio.

| *Ab*DGC vs *Ab*Pcob | | | | | | | |
| --- | --- | --- | --- | --- | --- | --- | --- |
| c-di-GMP produced (mM/min) | *Ab*DGC_only | (1 : 0.1) dark | (1 : 0.1) light | (1 : 1) dark | (1 : 1) light | (1 : 10) dark | (1 : 10) light |
| DGC activity 1 | 1.02 | 0.91 | 1.08 | 1.06 | 2.01 | 1.42 | 5.06 |
| DGC activity 2 | 0.98 | 0.95 | 1.10 | 1.17 | 2.15 | 1.48 | 5.04 |
| DGC activity 3 | 1.01 | 0.90 | 1.07 | 1.16 | 2.04 | 1.46 | 5.04 |

**Supplementary methods**

**Analytical gel filtration**

Full-length *Ab*DPcob protein was purified as detailed previously. Using a Superdex® 200pg 10/300 GL from Cytiva Life Sciences protein was analysed for changes to oligomerization state upon GTP binding. The column was equilibrated in 20mM HEPES, 150mM NaCl pH 8 before 0.3 mg protein was loaded. To expose *Ab*DPcob protein to light the protein was incubated for 30 minutes on ice in ambient light before loading. For GTP binding *Ab*DPcob protein was incubated with 250µM GTP (Sigma Aldrich) at 37°C 300rpm for 30 minutes before loading. The mass of the protein was determined from a calibration curve generated using a Gel Filtration Calibration Kit from Cytiva Life Sciences containing Ferritin (440,000 kDa), Aldolase (158,000 kDa), Conalbumin (75,000 kDa), Ovalbumin (44,000 kDa), Carbonic Anhidrase (29,000 kDa), Ribonuclaese A (13,700 kDa).

**Native mass spectrometry measurements of *Sas*Pcob and *Ab*DPcob**

On the day of analysis, following the manufacturer’s instructions protein was desalted into 200mM ammonium acetate pH 7.0 using Micro Bio-Spin 6 Chromatography columns (Bio-Rad). NanoESI capillaries were prepared in house from thin-walled borosilicate capillaries (inner diameter 0.9 mm, outer diameter 1.2 mm) (World Precision Instruments) using a Flaming/Brown P-97 micropipette puller (Sutter Instrument Company). Around 10µL of 5µM protein was used per capillary tube. Native MS data were acquired using the Thermo Scientific™ Q Exactive™ Hybrid Quadrupole-Orbitrap™ mass spectrometer (Thermo Fisher Scientific). A positive voltage was applied to the solution through a platinum wire (Goodfellow Cambridge Ltd., Huntington, UK) inserted into the capillary. For all spectra generated the spray current was kept between 0.2 and 0.3 µA with the spray voltage varying between 0.9-1.3 kV accordingly. The capillary temperature was kept constant at 350°C and the S-lens RF level at 200. The resolution used was 25,000 with 10 microscans and an AGC target of 1e^6^. The fore and ultra high vacuums were kept constant at 1.58e mbar and 2.15e^-10^ mbar respectively. Proteins were exposed to ambient light for 5 minutes before data collection. For analysis 5 minute spectra were averaged and processed in Thermo Xcalibur (Thermo Fisher Scientific).

**LC-MS and NMR analysis of Pcob proteins’ photo products**

To eliminate salt before the experiment protein was desalted using Micro Bio-Spin Chromatography columns (Bio-Rad, Micro Bio-Spin 6 Columns), following the manufacturer’s instructions with 200mM ammonium acetate pH 8.0. Samples were produced by using the same chromatography columns to separate 400µM protein from the eluent for 10 minutes in a microcentrifuge at 14,000rpm. For light exposed samples protein was left in ambient light for 10 minutes before centrifugation. 50µL of this mixture was used for LCMS whilst the remaining 250µL was diluted 1:2 with D2O (Sigma Aldrich) for Supplementary Figure 13. This was not sufficient for full elucidation and so the method was optimised for greater yields and less water contamination. 450mg CarH and 300mg SasPcob was illuminated with ambient light overnight. The photoproduct was separated using 10 KDa MWCO vivaspins (Sartorius). The flow through (~20-25mL) was dried on a Buchi Rotavapor R-300 at 30mbar 45°C 250rpm, resuspended in deuterated methanol and then dried again on a Genevac EZ-2 Mk3 using the low BP programme set at 45°C. The final powder was resuspended in 500µL deuterated methanol (Eurisotop). By changing protocol higher yields were generated with chemical shift values similar to those reported by Feng et al 2023^4^.

For LCMS the method optimised by Jost et al 2015 was utilised. Briefly, separation was undertaken on an Agilent 1100 LC-MSD instrument with a Agilent 150 x 3.0mm Poroshell 120 SB-C18 (2.7 µM pore size) reversed phase column using H2O with 0.1% v/v formic acid as solvent A and acetonitrile as solvent B. A linear gradient from 0% to 95% B was used at 8 minutes for 28 minutes followed by 100% B for 20 minutes then re-equilibration with 0% B for 15 minutes. Every 3-4 samples acetonitrile blanks were used to determine carry-over. An in-line ESI-TOF single quadrupole mass spectrometer (Agilent Technologies) in positive ion mode was used to determine mass-to-charge ratios (m/z). For the MS the following settings were used ESI capillary voltage 3000 V; gas temperature 350 °C; drying gas flow 11 L/min; nebulizer pressure 25 psi; fragmentor voltage 70 V; m/z scan range 120-1500.

NMR spectra were recorded on a Bruker AVIII 800 MHz AVIII spectrophotometer with a BBO probe equipped with Z-gradients. 1D 1H NMR spectra were at 298 K using a 1D 1H NMR method with presaturation water suppression (noesygppr1d, 256 scan, 2s acquisition time, 4s recycle/saturation delay 50Hz B1 saturation field strength). COSY spectra were CLIP-COSY^5^, ROESY are EASY-ROESY spectra with 200ms spinlock.

**Sequence similarity network of Pcob proteins**

The protein sequences were obtained from the BLASTP tools in Enzyme Function Initiative Tools - Enzyme Similarity Tool website (EFI-EST, <https://efi.igb.illinois.edu/efi-est/>). The Uniref40 IDs (648 IDs) of the protein sequences were used as inputs for further analysis. The SSN was downloaded and the key nodes was manually annotated, and then the network was visualized by Cytoscape V3.10.0 using force-direct layout.

**Protein function-oriented design of computational bioinformatic analysis**

Due to the lack of functional annotations of most Pcob-containing proteins in known databases and literature, our study utilized an integrated analysis based on multiple bioinformatic analyses and multidimensional information of Pcob-containing proteins such as sequence information, structural and functional information, genomic contextual information, and protein-protein interaction networks, to enhance the performance of the computational analysis and identification of protein functions. Based on the function-oriented design of the computational analysis, we were capable of inferring the common functions of the submitted protein sequence, and collectively, we might synthesize the functionality of Pcob-containing proteins.

**B_12_-binding domain** **homologous sequence searches, functional annotation and sequence similarity-based clustering**

We utilized the NCBI BLASTp algorithm (<https://blast.ncbi.nlm.nih.gov/Blast.cgi>) for the identification of B_12_-containing homologous sequence using B_12_-binding domain from CarH protein as the query sequence. 506 sequence was acquired and corresponding gene sequence information was collected for further analysis. Multiple sequence alignment (MSA) was performed based on all 506 Pcob-containing proteins using the MUSCLE algorithm. A phylogenetic tree using the neighborhood joining (NJ) method is constructed using Molecular Evolutionary Genetics Analysis software (version 10.2.6)^6^. All 506 BBD-containing proteins were mapped to Uniprot ID and submitted to the various database for preliminary annotation. The phylogenetic tree and related annotations are visualized by R using packages ggtree^7^ and ggtreeExtra^8^. The frequency of annotated terms was visualized by R package ggplot2, ggpubr, and Adobe Illustrator. To enhance the feature detection and reduce the statistical bias of downstream functional analysis, a multi-step sequence similarity-based clustering was performed using h-cd-hit^9^ (Threshold: 0.9, 0.6, 0.3). Representative sequences of clustering results (37 clusters) were used for further analysis.

**Protein structure prediction, comparison, and analysis**

Sequence alignment of 37 representative protein sequences was performed using ClustalW (<https://www.genome.jp/tools-bin/clustalw>). The three-dimensional protein structure was predicted by AlphaFold2^10^, an accurate computational approach to predict the three-dimensional structure of target proteins based on deep learning algorithms. Alignment results and The 3D structure of *Sas*Pcob were submitted to ESPript (Version 3.0, <https://espript.ibcp.fr/ESPript/cgi-bin/ESPript.cgi>) for structural annotation^11^. MSA alignment and structural annotation results were visualized by R package ggmsa^12^ and Adobe illustrator.

Motif prediction was performed based on MEME Suite^13^, a well-known tool for the discovery of novel, ungapped motifs. Protein domain prediction was performed using the simple modular architecture research tool (SMART)^14^ and NCBI Conserved Domain Database (CDD)^15^. The phylogenetic tree and architecture of predicted motifs and domains were collected and visualized by Adobe illustrator and TBtools^16^, a platform for integrative analyses and visualization of big biological data.

**Supplementary discussion**

**Analysis of *Ab*Pcob MD simulation and BV ensemble docking results**

To explore the possible binding pose with BV, docking was performed on representative structures of the MD trajectories from clustering of dark-state *Ab*Pcob and *AbD*Pcob simulations. Structure distributions for different clusters are displayed in Tables S1 and S5. Maximum binding affinities of BV docking results reveal that around 80% of the conformations of *Ab*Pcob showed predicted binding affinity over 0, indicating non-favourable binding to BV. Only 4% of the *Ab*Pcob conformations could confidently bind BV with binding affinity lower than -7.0 kcal/mol. Among the rest, some could retain BV in the protein, but the interaction between BV and protein are not strong enough. For *AbD*Pcob, the structures are stable after the simulated annealing runs (**Supplementary** **figure** **7**), and in this case 80 % of structures bind BV with a binding affinity below -7.0 kcal/mol (examples are shown in **Supplementary** **figure** **8**). In the light state, a much larger RMSD is observed during one of these runs (Anneal c in **Supplementary Figure 7**), which is due to a repositioning of the DGC domain, not increased structural instability during the simulation: root-mean-squared fluctuations (RMSF) were calculated for each individual run (not including the initial 50 ns annealing), so that conformational change between runs would not affect the result, and the RMSF of each run is very similar, indicating no significant change in the magnitude of the motions. Crucially, this suggests that using MD simulations to rearrange the protein is sufficient for BV binding in certain conformations.

**Sequence similarity network of Pcob proteins**

*Sas*Pcob protein sequence was used as a query in BLASTP analysis to identify all *Sas*Pcob-related sequences. A representative sequence from the Uniref40 database was selected for further analysis. The sequences were then clustered based on their sequence similarity using the force-directed graph layout. The resulting two-dimensional graph (**Supplementary** **figure** **26**) exhibited five distinct clusters, with *Sas*Pcob, CarH, and Methionine synthase grouped together in one cluster. The remaining four clusters were clearly delineated and represented by Methyltetrahydrofolate, long-chain-fatty-acid-CoA ligase, Glycosytransferase, and Phosphoglycerate dehydrogenase sequences.

**Distribution analysis and functional prediction of Pcob proteins**

A total of 506 photocobilin photoreceptor proteins with a high similarity of overall sequence, structure, motifs and domain architecture were identified, either fused to other enzyme domains or as standalone proteins (**Supplementary Figure 27**). To explore the possible biological function of photocobilin proteins we used bioinformatics approaches to explore their potential cellular roles. A multilevel bioinformatic analysis pipeline based on multidimensional information (features identification, genomic contextual information, and protein-protein interaction) of photocobilin containing proteins has been designed and utilised (**Supplementary Figure 27-30**). 506 Pcob proteins have been clustered into 37 groups based on their sequence and evolution relationship (**Supplementary Figure 27-29**). The results of the phylogenetic analysis demonstrate that Pcob proteins are widely distributed across various types of microorganisms, ranging from archaea to bacteria. Notably, a majority of the Pcobs are found in the terrabacteria group, which is known for its crucial adaptations including resistance to environmental hazards and the ability to carry out oxygenic photosynthesis^17^. The evolutionary trajectory of the fusion proteins suggests they have evolved over time, implying that the close proximity of the output domain is likely to be a more efficient mode of regulation. A number of common output domains are found to be fused to, and thus regulated by, photocobilin domains, including transcriptional regulators, DNA polymerase and serine/threonine phosphatase (**Supplementary Figure 30**). Standalone photocobilin proteins are frequently associated with transcriptional regulators (e.g. TetR family), transporters (e.g. ABC transporter) and many domains of prokaryotic signal transduction systems (e.g., histidine kinase, histidine phosphotransferase, diguanylate cyclase, PAS/PAC sensor hybrid histidine kinase, SpoIIE and STAS domains).

**Comparison of predicted Pcob structures with *Sas*Pcob**

Pcob proteins have been clustered into 37 groups. Represented Pcob protein structures were downloaded from AlphaFold2 database^1^. Comparison of structural alignment with *Sas*Pcob revealed that Pcobs have similar structure arrangement with either chromophore placed at N-terminal (**Supplementary Figure 31**). Although different Pcobs have low sequence identity, their predicted 3D structure are looks like the same, RMSD value of C-alpha atoms ranging from 1.250 to 2.775. The linker region affects the relative position of two chromophores, which might be the key for investigating the function of Pcobs during the evolution.

**Supplementary references**

1. Varadi M*, et al.* AlphaFold Protein Structure Database: massively expanding the structural coverage of protein-sequence space with high-accuracy models. *Nucleic Acids Res* **50**, D439-d444 (2022).

2. Jumper J*, et al.* Highly accurate protein structure prediction with AlphaFold. *Nature* **596**, 583-589 (2021).

3. Brown NP, Leroy C, Sander C. MView: a web-compatible database search or multiple alignment viewer. *Bioinformatics* **14**, 380-381 (1998).

4. Feng X, Zhang Q, Clarke DJ, Deng H, O'Hagan D. 3'-O-beta-Glucosyl-4',5'-didehydro-5'-deoxyadenosine Is a Natural Product of the Nucleocidin Producers Streptomyces virens and Streptomyces calvus. *J Nat Prod* **86**, 2326-2332 (2023).

5. Koos MR, Kummerlowe G, Kaltschnee L, Thiele CM, Luy B. CLIP-COSY: A Clean In-Phase Experiment for the Rapid Acquisition of COSY-type Correlations. *Angew Chem Int Ed Engl* **55**, 7655-7659 (2016).

6. Kumar S, Stecher G, Li M, Knyaz C, Tamura K. MEGA X: Molecular Evolutionary Genetics Analysis across Computing Platforms. *Molecular biology and evolution* **35**, 1547-1549 (2018).

7. Yu G, Smith DK, Zhu H, Guan Y, Lam TT-Y. ggtree: an r package for visualization and annotation of phylogenetic trees with their covariates and other associated data. **8**, 28-36 (2017).

8. Xu S*, et al.* ggtreeExtra: Compact Visualization of Richly Annotated Phylogenetic Data. *Molecular biology and evolution* **38**, 4039-4042 (2021).

9. Huang Y, Niu B, Gao Y, Fu L, Li W. CD-HIT Suite: a web server for clustering and comparing biological sequences. *Bioinformatics (Oxford, England)* **26**, 680-682 (2010).

10. Jumper J*, et al.* Highly accurate protein structure prediction with AlphaFold. *Nature* **596**, 583-589 (2021).

11. Robert X, Gouet P. Deciphering key features in protein structures with the new ENDscript server. *Nucleic acids research* **42**, W320-W324 (2014).

12. Zhou L*, et al.* ggmsa: a visual exploration tool for multiple sequence alignment and associated data. *Briefings in bioinformatics*, (2022).

13. Bailey TL, Johnson J, Grant CE, Noble WS. The MEME Suite. *Nucleic acids research* **43**, W39-49 (2015).

14. Letunic I, Khedkar S, Bork P. SMART: recent updates, new developments and status in 2020. *Nucleic acids research* **49**, D458-D460 (2020).

15. Lu S*, et al.* CDD/SPARCLE: the conserved domain database in 2020. *Nucleic acids research* **48**, D265-d268 (2020).

16. Chen C*, et al.* TBtools: An Integrative Toolkit Developed for Interactive Analyses of Big Biological Data. *Molecular plant* **13**, 1194-1202 (2020).

17. Battistuzzi FU, Hedges SB. A major clade of prokaryotes with ancient adaptations to life on land. *Mol Biol Evol* **26**, 335-343 (2009).
