# Supplementary material for: Photocobilins integrate B12 and bilin photochemistry for enzyme control
Source: Nat Commun. 2024 Mar 28;15:2740. doi: 10.1038/s41467-024-46995-1 (PMC10979010; doi:10.1038/s41467-024-46995-1)
Supplement: Supplementary file 3 — Description of Additional Supplementary Files [file 41467_2024_46995_MOESM3_ESM.pdf]

Description of Additional Supplementary Files

File Name: Supplementary Data 1

Description: Clustered *Ab*Pcob structure after 500ns MD simulation.

File Name: Supplementary Movie 1

Description: Molecular dynamics simulation of *Ab*DPcob dark structure.

1500 ns simulation was carried out for *Ab*DPcob dark structure. *Ab*DPcob Protein is shown as cartoon. B_{12} domain is shown as lemon-green, BV as light-pink and DGC as light-blue. The linker regions are shown as grey.

File Name: Supplementary Movie 2

Description: Molecular dynamics simulation of *Ab*DPcob light structure.

1500 ns simulation was carried out for *Ab*DPcob light structure. *Ab*DPcob Protein is shown as cartoon. B_{12} domain is shown as green, BV as hot-pink and DGC as marine-blue. The linker regions are shown as grey.
