# Supplementary material for: Photocobilins integrate B12 and bilin photochemistry for enzyme control
Source: Nat Commun. 2024 Mar 28;15:2740. doi: 10.1038/s41467-024-46995-1 (PMC10979010; doi:10.1038/s41467-024-46995-1)
Supplement: Supplementary file 7 — Reporting Summary [file 41467_2024_46995_MOESM7_ESM.pdf]

nature portfolio

Corresponding author(s): *Double-blind peer review submissions: write DBPR and your manuscript number here instead of author names.*

Last updated by author(s): *YYYY-MM-DD*

Reporting Summary

Nature Portfolio wishes to improve the reproducibility of the work that we publish. This form provides structure for consistency and transparency in reporting. For further information on Nature Portfolio policies, see our Editorial Policies and the Editorial Policy Checklist.

Statistics

For all statistical analyses, confirm that the following items are present in the figure legend, table legend, main text, or Methods section.

n/a	Confirmed	
☐	☒	The exact sample size (n) for each experimental group/condition, given as a discrete number and unit of measurement
☒	☐	A statement on whether measurements were taken from distinct samples or whether the same sample was measured repeatedly
☐	☒	The statistical test(s) used AND whether they are one- or two-sided<br>*Only common tests should be described solely by name; describe more complex techniques in the Methods section.*
☒	☐	A description of all covariates tested
☒	☐	A description of any assumptions or corrections, such as tests of normality and adjustment for multiple comparisons
☐	☒	A full description of the statistical parameters including central tendency (e.g. means) or other basic estimates (e.g. regression coefficient) AND variation (e.g. standard deviation) or associated estimates of uncertainty (e.g. confidence intervals)
☐	☒	For null hypothesis testing, the test statistic (e.g. F, t, r) with confidence intervals, effect sizes, degrees of freedom and P value noted<br>*Give P values as exact values whenever suitable.*
☒	☐	For Bayesian analysis, information on the choice of priors and Markov chain Monte Carlo settings
☒	☐	For hierarchical and complex designs, identification of the appropriate level for tests and full reporting of outcomes
☒	☐	Estimates of effect sizes (e.g. Cohen's d, Pearson's r), indicating how they were calculated

Our web collection on statistics for biologists contains articles on many of the points above.

Software and code

Policy information about availability of computer code

Data collection	Crystal diffraction data was collected at Diamond Light Source beamlines, using GDA. UV-Vis spectroscopy data was collected using Agilent Cary Win UV software. MS data was collected using Agilent or Thermo software for photoproduct determination and native MS respectively. NMR data was collected using Bruker TopSpin.
Data analysis	COOT 0.9.8.8, CCP4 8.0.013, Phaser MR 2.8.3, Pymol 2.5, EasySpin toolbox 5.2.28, Origin Pro 9.1,GraphPad Prism 9.0, PHENIX 1.20.1- 4487, PDBredo, PDBeFOLD, PDB2PQR 2.1.0, ABPS server, ChimeraX 1.3, Thermo Xcalibur, Agilent MassHunter, UniDec, Bruker TopSpin, MestreNova, Inkscape

For manuscripts utilizing custom algorithms or software that are central to the research but not yet described in published literature, software must be made available to editors and reviewers. We strongly encourage code deposition in a community repository (e.g. GitHub). See the Nature Portfolio guidelines for submitting code & software for further information.

Data

Policy information about availability of data

All manuscripts must include a data availability statement. This statement should provide the following information, where applicable:

- Accession codes, unique identifiers, or web links for publicly available datasets
- A description of any restrictions on data availability
- For clinical datasets or third party data, please ensure that the statement adheres to our policy

The atomic coordinates and experimental data generated in this study have been deposited in the Protein Data Bank (www.pdb.org) under accession code: 8J2W [https://www.rcsb.org/structure/8J2W], 8J2X [https://www.rcsb.org/structure/8J2X], 8J2Y [https://www.rcsb.org/structure/8J2Y]. Atomic coordinates referenced in the main text can be found through 1B33 [https://www.rcsb.org/structure/1B33], 3MWN (https://www.rcsb.org/structure/3MWN). SAXS data has been deposited in the small Angle Scattering Biological Data Bank (www.sasbdb.org) under accession codes SASDUS3 (www.sasbdb.org/data/SASDUS3/) and SASDUT3 (www.sasbdb.org/data/SASDUT3/). LCMS data has been deposited on Figshare (Jeffreys, Laura [2024]). Photoproduct determination - LCMS. Figshare. Journal contribution. https://doi.org/10.6084/m9.figshare.25219721). LCMS data has been deposited on Figshare (Jeffreys, Laura (2024). Photoproduct determination - NMR. Figshare. Journal contribution. https://doi.org/10.6084/m9.figshare.25219832). MD parameters for AdoCbl and OHCbl have been deposited on Figshare (https://doi.org/10.6084/m9.figshare.25226480). Initial and final structures from MD simulations have been deposited on Figshare (https://doi.org/10.6084/m9.figshare.25226549). All other source data are provided as supplementary data files. Source data are provided in this paper.

Human research participants

Policy information about studies involving human research participants and Sex and Gender in Research.

Reporting on sex and gender	N/A
Population characteristics	N/A
Recruitment	N/A
Ethics oversight	N/A

Note that full information on the approval of the study protocol must also be provided in the manuscript.

Field-specific reporting

Please select the one below that is the best fit for your research. If you are not sure, read the appropriate sections before making your selection.

☒ Life sciences ☐ Behavioural & social sciences ☐ Ecological, evolutionary & environmental sciences

For a reference copy of the document with all sections, see nature.com/documents/nr-reporting-summary-flat.pdf

Life sciences study design

All studies must disclose on these points even when the disclosure is negative.

Sample size	Samples were performed in typical triplicate where necessary.
Data exclusions	There were no data exclusions
Replication	Samples were performed in triplicate where necessary including modelling, UV-Vis and enzymatic assays.
Randomization	Randomisation was not applicable for the methods used
Blinding	Blinding was not applicable for our samples as no human bias was probable

Reporting for specific materials, systems and methods

We require information from authors about some types of materials, experimental systems and methods used in many studies. Here, indicate whether each material, system or method listed is relevant to your study. If you are not sure if a list item applies to your research, read the appropriate section before selecting a response.

Materials & experimental systems

n/a	Involved in the study
☒	☐ Antibodies
☒	☐ Eukaryotic cell lines
☒	☐ Palaeontology and archaeology
☒	☐ Animals and other organisms
☒	☐ Clinical data
☒	☐ Dual use research of concern

Methods

n/a	Involved in the study
☒	☐ ChIP-seq
☒	☐ Flow cytometry
☒	☐ MRI-based neuroimaging
